# Polyphenolic Composition and Multifunctional Biological Properties of Raspberry Leaves: Variation Among Cultivar-Specific Samples

**DOI:** 10.3390/molecules31193419

**Published:** 2026-09-25

**Authors:** Marcelina Stach, Dorota Bęben, Helena Moreira, Joanna Kolniak-Ostek

**Affiliations:** 1 Department of Fruit, Vegetable and Plant Nutraceutical Technology, Wrocław University of Environmental and Life Sciences, 37 Chełmońskiego Street, 51-630 Wroclaw, Poland; marcelina.stach@upwr.edu.pl; 2 Department of Basic Medical Sciences, Faculty of Pharmacy, Wroclaw Medical University, 211 Borowska Street, 50-556 Wroclaw, Poland; dorota.beben@student.umw.edu.pl (D.B.); helena.moreira@umw.edu.pl (H.M.)

**Keywords:** raspberry, UPLC–Q/TOF-MS, polyphenols, antioxidant capacity, enzyme inhibition, cyclooxygenase inhibition, colorectal cancer cell viability

## Abstract

Despite increasing interest in raspberry leaves (*Rubus idaeus* L.) as a source of bioactive compounds, comparative information linking their phenolic composition to multiple biological properties remains limited. This study compared the polyphenolic profiles and biological properties of leaves from seven raspberry cultivars representing *Rubus idaeus* L. and *Rubus occidentalis* L., grown under identical environmental conditions. Phenolic compounds were characterized by UPLC–Q/TOF-MS, while antioxidant capacity, α-amylase and α-glucosidase-inhibitory activities, inhibition of COX-1 and COX-2, and effects on colorectal cancer cell viability were evaluated. A total of 37 phenolic compounds were identified or tentatively characterized, with flavan-3-ols and procyanidins representing the quantitatively predominant phenolic group, followed by flavonols. Considerable variation in both phenolic composition and biological responses was observed among the cultivar-specific composite samples. Red-fruited samples generally showed higher antioxidant capacity, whereas α-amylase- and α-glucosidase-inhibitory activities varied among the samples. In contrast, the strongest COX-1 and COX-2 inhibition was observed for the yellow-fruited Poranna Rosa sample. Effects on colorectal cancer cell viability also varied among the samples, with the red-fruited samples generally producing greater reductions in LOVO, LOVO-DX, and SW48 cell viability. Exploratory Spearman correlation analysis using exact permutation-based *p*-values followed by FDR correction showed that flavan-3-ol and total polyphenol concentrations were positively associated with ABTS and FRAP antioxidant capacity and inversely associated with LOVO, LOVO-DX and SW48 cell viability at 600 μg/mL. No FDR-significant associations were observed for carbohydrate-hydrolyzing enzyme or COX inhibition. Overall, these results demonstrate substantial variation in the phenolic composition and biological properties among the analyzed raspberry leaf samples analyzed. Because the species, cultivar identity, and fruit color group cannot be separated within the present experimental design, the observed differences should not be independently attributed to genotype or fruit color. These findings support further investigation of raspberry leaves as sources of bioactive phenolic compounds for potential food and nutraceutical applications.

## 1. Introduction

*Rubus idaeus* L. (red raspberry) is widely cultivated throughout Europe, North America, and Asia, and its fruits are among the most popular berries consumed worldwide in fresh, frozen, and processed forms [1,2]. Intensive raspberry production and industrial processing generate considerable amounts of agricultural by-products, including stems, seeds, and leaves. Among these materials, raspberry leaves have attracted increasing scientific interest because they are a rich source of phytochemicals that can be used in the development of functional foods, nutraceuticals, cosmetic formulations, and pharmaceutical products, thus contributing to the more sustainable use of plant resources [3,4].

The increasing prevalence of noncommunicable diseases, particularly obesity, diabetes, and other metabolic disorders, has intensified the search for plant-derived ingredients with scientifically documented health-promoting properties. Polyphenol-rich plant materials are considered important dietary sources of bioactive compounds that can help reduce oxidative stress and lower the risk of chronic diseases [5,6]. Although raspberry fruits have been extensively investigated in this context, increasing attention has recently been paid to raspberry leaves, which often contain higher concentrations of phenolic constituents and therefore represent an attractive source of biologically active compounds [7].

Numerous studies have shown that raspberry leaves exhibit a broad spectrum of biological activities, largely attributed to their phenolic composition [8,9,10]. Their extracts possess strong antioxidant capacity, protect cells against oxidative damage, and have been reported to exhibit anti-inflammatory, antidiabetic, antibacterial, and other pharmacological properties. Traditionally, raspberry leaves have also been used in the treatment of fever, influenza, diarrhea, glaucoma, and during labor, highlighting their long-standing importance in herbal medicine [11]. These biological effects are associated with the presence of various phenolic compounds, including hydroxybenzoic and hydroxycinnamic acids, flavonoids, derivatives of ellagic acid, tannins, and other specialized metabolites in the leaves. In addition to polyphenols, raspberry leaves contain minerals, sterols, organic acids, mucilage, and resinous compounds, which further contribute to their nutritional and therapeutic value [12,13].

Beyond their nutritional significance, raspberry leaves are a valuable source of naturally occurring compounds with antimicrobial and preservative properties, making them promising ingredients for applications in the food, cosmetic, and pharmaceutical industries. Plant-derived phenolics have long served as a source of inspiration in the development of therapeutic agents, with aspirin being one of the best-known examples of a synthetic drug developed from the naturally occurring phenolic compound salicylic acid [14].

The composition of phenolic compounds in raspberry leaves is influenced by numerous factors, including genotype, cultivar, environmental conditions, and developmental stage [15]. Genetic and environmental factors have also been associated with variation in the pigmentation of raspberry fruit and the phenolic composition [16,17]. However, considerably less is known about the extent of variation in leaf phenolic composition and biological properties between raspberry cultivars representing different groups and fruit-color species. Comparative characterization of this plant material under common cultivation and analytical conditions may help identify compositional and biological differences among the investigated samples while reducing the variation associated with environmental and methodological factors. However, because fruit color, cultivar identity, and species may be interrelated within a given set of plant material, such comparisons do not necessarily allow their individual contributions to be distinguished.

Therefore, the present study aimed to comprehensively characterize the polyphenolic composition of leaves collected from seven raspberry cultivars representing red, yellow, and black-fruited plant material of *R. idaeus* and *R. occidentalis* using UPLC-PDA-Q/TOF-MS analysis. Furthermore, antioxidant capacity, α-amylase- and α-glucosidase-inhibitory activity, inhibition of COX-1 and COX-2 inhibition, and effects on colorectal cancer cell viability were evaluated to provide an integrated comparison of the phytochemical and biological properties of the analyzed raspberry leaf samples. We hypothesized that the investigated cultivar-specific leaf samples would differ in their polyphenolic profiles and that variation in phenolic composition would be associated with selected biological responses.

## 2. Results and Discussion

### 2.1. Polyphenolic Profile of Raspberry Leaves

The detailed UPLC–PDA–Q/TOF-MS profile revealed pronounced qualitative and quantitative variation in the phenolic composition of the analyzed cultivar-specific composite raspberry leaf samples. Although all samples analyzed shared the same major classes of phenolic compounds, including phenolic acids, flavan-3-ols, flavonols, flavones, and ellagic acid derivatives, their relative abundances varied considerably among the samples, resulting in distinct phenolic profiles. In total, 37 phenolic compounds were identified or tentatively characterized (Table 1), with flavan-3-ols, procyanidins, and flavonols representing the quantitatively predominant phenolic classes.

Total phenolic concentrations varied considerably among the analyzed cultivar-specific composite samples. The red-fruited raspberry cultivars exhibited the highest total phenolic concentrations (67.3–69.5 g/kg DW), followed by black raspberry cultivars (56.2–56.5 g/kg DW), while the yellow-fruited cultivars showed the lowest values (49.4–53.0 g/kg DW). These quantitative differences were accompanied by marked variation in the relative contribution of individual phenolic classes, indicating that the analyzed samples differed not only in total phenolic concentration, but also in the distribution of phenolic subclasses.

Although they shared the same main classes of phenolic metabolites, the analyzed cultivar-specific composite samples exhibited marked variation in the relative distribution of individual compounds. The higher total phenolic concentration observed in red fruit cultivars primarily reflected the high abundance of flavan-3-ols and procyanidins, which represented more than half of the quantified phenolic content. On the contrary, black raspberry leaves contained proportionally higher levels of flavonols together with selected derivatives of hydroxycinnamic acid, while yellow fruit cultivars displayed a more balanced phenolic profile, characterized by a lower proportion of flavan-3-ols and relatively greater contributions of low molecular weight phenolic acids and flavones. Therefore, the variation observed among the analyzed samples involved differences in the relative distribution of multiple phenolic subclasses rather than changes restricted to individual metabolites. Such compositional patterns may be related to differences in the regulation of phenylpropanoid metabolism; however, the present experimental design did not directly assess biosynthetic pathway activity or its genetic regulation. Further studies incorporating independent biological replication and targeted analysis of the regulation of the phenylpropanoid pathway would be required to determine the mechanisms underlying the observed differences in phenolic composition.

In addition to the variation in the abundance of the main phenolic classes, several metabolites showed distinct distribution patterns among the analyzed cultivar-specific composite samples. Within the present sample set, epicatechin was detected only in the three red-fruited cultivar samples, whereas quercetin *O*-rutinoside was detected only in the two black raspberry samples. Caffeic acid *O*-hexoside isomer 4 was particularly abundant in black raspberry samples, reaching concentrations markedly higher than those observed in the yellow-fruited samples, while it was not detected in the red-fruited samples. In contrast, quinic acid was detected in the yellow- and black-fruited samples but not in the red fruits, while apigenin *O*-pentoside occurred at comparatively high concentrations in the yellow-fruited samples. Collectively, these observations indicate that the composite samples differed not only in the abundance of the main phenolic classes but also in the distribution of individual metabolites.

Recent advances have demonstrated that proanthocyanidin accumulation is controlled by a complex regulatory network that involves the phenylpropanoid pathway, including chalcone synthase (CHS), dihydroflavonol reductase (DFR), anthocyanidin synthase (ANS), anthocyanidin reductase (ANR), and leucoanthocyanidin reductase (LAR). In addition to enzyme activities, metabolic channeling, intracellular transport, and transcriptional regulation contribute to genotype-dependent differences in flavan-3-ol accumulation [18]. In raspberry leaves, Kobori et al. [19] demonstrated that increased accumulation of catechin, epicatechin and procyanidins was accompanied by increased CHS expression, supporting the transcriptional regulation in flavan-3-ol accumulation. These findings provide a plausible biochemical context for the differences in the abundance of flavan-3-ol and procyanidin observed among the composite samples analyzed in the present study. However, because the expression or activity of enzymes involved in phenylpropanoid and flavonoid biosynthesis was not evaluated here, the metabolic mechanisms underlying these compositional differences cannot be established from the present data.

The phenolic composition observed in the present study is consistent with previous reports that identify raspberry leaves as rich sources of flavan-3-ols, flavonols, phenolic acids and ellagic acid derivatives [20,21,22]. Similar LC–MS-based investigations have demonstrated that these compound classes constitute the dominant phenolics in raspberry leaves, although both the number of detected metabolites and their relative abundances vary considerably between studies. Such differences may result from multiple factors, including plant genotype, geographical origin, environmental conditions, development stage, and harvest time, as well as differences in extraction procedures and analytical platforms [5,12]. In particular, Pavlovi et al. [23] reported substantial cultivar-dependent variation in the relative abundance of ellagitannins, flavonols and flavan-3-ols, emphasizing that genetic background is one of the principal determinants of the phenolic composition of raspberry leaves.

In the present study, the environmental and methodological sources of variation were reduced by cultivating all plants at the same experimental site under comparable agronomic conditions, collecting leaves at the same stage and on the same sampling date, and applying identical sample preparation and analytical procedures. This controlled sampling strategy strengthens the comparability of the phenolic profiles obtained for the analyzed cultivar-specific composite samples and reduces the variation attributable to differences in the cultivation site, harvest time, and analytical methodology. However, because only one pooled composite biological sample was analyzed per cultivar, the present study does not allow the estimation of biological variability to be estimated or the observed differences to be unequivocally attributed to cultivar or genotype.

An additional limitation arises from the taxonomic structure of the investigated material. The red- and yellow-fruited cultivars belong to *Rubus idaeus* L., while the two black raspberry cultivars belong to *Rubus occidentalis* L. Consequently, the identity of the species is confounded with the black-fruited group, and the effects of the species, cultivar/genotype, and fruit color group cannot be separated statistically within the present experimental design. Therefore, the observed phenolic patterns should be interpreted as compositional differences among the cultivar-specific composite samples analyzed rather than as independent effects of genotype or fruit color. Within *R. idaeus*, the red- and yellow-fruited samples also showed differences in both total phenolic concentration and the relative distribution of the main phenolic classes; however, the limited number of cultivars and the absence of independent biological replication preclude the generalization of these observations to fruit-color groups. More studies including a larger number of cultivars within each species and fruit color category, together with independent biological replication, will be required to unravel the relative contributions of genotype, species, and fruit color to the phenolic composition of the raspberry leaf.

The revised phytochemical profile also emphasizes the importance of considering identification confidence when interpreting the phenolic composition of raspberry leaves. Among the 37 compounds retained after re-evaluation, gallic acid, caffeic acid neochlorogenic acid, and (+)-catechin were confirmed by comparison with authentic standards (Level 1), while the remaining constituents were identified or tentatively characterized according to accurate mass, MS/MS fragmentation, and evidence from the literature (Levels 2–3). From a quality-control perspective, the consistent occurrence of selected constituents across all analyzed samples suggests their potential utility in a multimarker approach to standardizing raspberry leaf preparations. In particular, (+)-catechin, A-type procyanidin trimer, and quercetin *O*-pentoside isomer 2 may be considered candidate markers because they were detected in all seven samples and represent the main constituents of the quantitatively predominant flavan-3-ol/procyanidin and flavonol fractions. A multimarker strategy may provide a more representative assessment of phytochemical consistency than reliance on a single constituent, particularly given the differences in phenolic profiles observed among the raspberry types investigated. However, the present study was not designed to assess batch-to-batch reproducibility or the stability of the preparations during processing and storage. Therefore, the suitability of these compounds as quality control markers, including their concentration ranges and stability, should be validated using batches produced independently obtained from different harvests and subjected to defined processing and storage conditions before formal standardization criteria can be established.

### 2.2. Biological Activity

The antioxidant capacity of the raspberry leaf extracts varied among the cultivar-specific composite samples, as demonstrated by the ABTS, DPPH and FRAP assays (Table 2).

Among the samples analyzed, the red-fruited cultivar samples generally showed higher ABTS, DPPH and FRAP values, whereas lower values were observed for the yellow-fruited samples and intermediate values for the black raspberry samples. This variation in antioxidant capacity was accompanied by differences in the phenolic composition between the samples analyzed, particularly in the abundance and relative distribution of the major phenolic classes. The potential relationships between phenolic composition and antioxidant capacity are further examined below using exploratory correlation analysis.

The high antioxidant potential of raspberry leaves is generally attributed to the presence of phenolic constituents, including hydrolyzable tannins, particularly ellagitannins, flavan-3-ols, procyanidins and flavonol glycosides, which are among the dominant classes of phenolic compounds in *Rubus* leaves. Among the compounds identified in the present study, catechin derivatives, procyanidins, and quercetin glycosides are recognized as effective antioxidants due to their ability to donate hydrogen atoms or electrons, stabilize free radicals, and chelate transition metal ions. The relatively high abundance of these compounds in samples showing greater antioxidant capacity therefore provides a plausible biochemical basis for the observed pattern, although their individual contributions cannot be established from the present data. Previous studies have also reported associations between the phenolic composition of raspberry leaves and their antioxidant capacity. For example, Pavlović et al. [23] reported variation in both phenolic profiles and antioxidant properties between leaf extracts from different raspberry and blackberries.

The relationship between phenolic composition and antioxidant capacity observed in the present study is consistent with previous studies on raspberry leaves. Ponder and Hallmann [12] reported differences in both phenolic composition and antioxidant capacity between leaves of various cultivars of *R. idaeus* and observed a significant relationship between total polyphenol content and in vitro antioxidant activity. Similarly, Varzaru et al. [24] demonstrated a substantial antioxidant capacity of raspberry leaves using several complementary in vitro assays, including DPPH, ABTS, and hydroxyl radical scavenging assays, along with a high content of total polyphenols and flavonoids. Collectively, these studies support an association between the abundance of phenolic constituents and the antioxidant properties of raspberry leaves, while also indicating that the magnitude of antioxidant responses may vary depending on the phenolic composition and the assay employed.

Although all three antioxidant assays demonstrated substantial antioxidant capacity in raspberry leaf extracts, slight differences in cultivar classification were observed between ABTS, DPPH, and FRAP. Such variation is expected because these assays assess different aspects of antioxidant capacity and differ in their reaction mechanisms and sensitivity to individual antioxidant compounds. DPPH and ABTS assess primarily radical scavenging capacity through hydrogen atom or electron transfer reactions, while FRAP evaluates the ferric ion-reducing capacity of antioxidants. Therefore, antioxidant capacity depends not only on the total abundance of phenolic compounds but also on their molecular structure, redox properties, and relative proportions within the extract. Therefore, the structural diversity of the phenolic components detected in the raspberry leaf extracts, including flavan-3-ols, procyanidins, ellagic acid derivatives, and flavonol derivatives, may contribute to the differences in antioxidant responses observed between the analyzed samples and between the three assays.

Furthermore, the comprehensive characterization of the phenolic constituents presented by Alkhudaydi et al. [22] confirmed that raspberry leaves are particularly rich in ellagitannins, flavan-3-ols, and flavonol derivatives, which collectively contribute to their high antioxidant potential and biological value.

The present results also support previous reports indicating that raspberry leaves are a rich source of antioxidant phytochemicals compared to several other aerial-plant materials. Zielonka-Brzezicka et al. [25] demonstrated that raspberry leaf extracts exhibited consistently high antioxidant activity in the ABTS, DPPH and FRAP assays, confirming that leaves are a valuable source of antioxidant compounds within raspberry plants. Collectively, these findings reinforce the growing evidence that raspberry leaves, traditionally considered an agricultural by-product, constitute an attractive and sustainable source of natural antioxidants for the development of functional foods, nutraceuticals, and other value-added products.

Inflammation is a complex biological response that involves the activation of numerous signaling pathways and inflammatory mediators. Cyclooxygenases (COX-1 and COX-2) catalyze the conversion of arachidonic acid to prostaglandin precursors and represent important molecular targets in modulation of inflammatory processes. Therefore, inhibition of COX activity is commonly used as one of the in vitro indicators of the potential of bioactive compounds to modulate pathways associated with inflammation. In the present study, all raspberry leaf extracts inhibited both COX-1 and COX-2; however, considerable differences were observed among the cultivar-specific composite samples (Table 2). The highest inhibitory activity against both cyclooxygenase isoforms was recorded for the yellow raspberry cultivar Poranna Rosa, while the lowest activity was observed for Glen Ample. These results demonstrate variation in COX-inhibitory activity among the analyzed raspberry leaf samples; however, this endpoint alone should not be interpreted as evidence of general anti-inflammatory activity.

Previous studies have identified raspberry leaves as a source of phenolic compounds with the potential to modulate inflammatory processes, including ellagitannins, flavan-3-ols, flavonols, and phenolic acids. These compounds have been reported to affect multiple inflammation-related targets and pathways, including cyclooxygenase and lipoxygenase activity, cellular antioxidant defense systems, and pro-inflammatory signaling pathways [26]. Such effects are likely to reflect the combined contributions of multiple phenolic components rather than the activity of a single dominant compound, as also discussed in recent reviews of the biological properties of raspberry leaves and other plant materials. Interestingly, the pattern of COX inhibition did not parallel the total phenolic concentration or the abundance of the dominant flavan-3-ol and procyanidin fractions in the samples analyzed (Table 1 and Table 2). Although red-fruited samples generally contained higher concentrations of flavan-3-ols and procyanidins than yellow-fruited samples, the strongest inhibition of both COX-1 and COX-2 was observed for Poranna Rosa, which had a lower total phenolic concentration than the red-fruited samples. On the contrary, Glen Ample, despite its high total phenolic concentration and abundance of flavan-3-ols and procyanidins, showed the lowest inhibition of both cyclooxygenase isoforms. These observations indicate that the total phenolic concentration alone does not account for the pattern of COX inhibition observed in the analyzed raspberry leaf samples. Differences in the relative abundance of individual phenolic constituents, as well as possible additive or interactive effects within complex extracts, may contribute to the observed COX-inhibitory activity; however, the contributions of individual compounds and their interactions were not directly assessed in the present study.

Previous studies provide complementary evidence that raspberry leaf constituents can modulate inflammation-related processes. Maslov et al. [27] demonstrated that raspberry leaf extracts significantly reduced carrageenan-induced inflammation in vivo and suggested that this activity was associated with the presence of catechins, ellagic acid derivatives, and other bioactive phenolics that act synergistically rather than independently. Luo et al. [28] also reported variation in phenolic composition and inflammation-related activities in vitro among a diverse set of raspberry leaf samples, although their evaluation was based on hyaluronidase and inhibition of albumin denaturation rather than cyclooxygenase activity. Together, these studies indicate that the potential of raspberry leaf extracts to modulate inflammation-related processes may involve multiple phenolic constituents and may vary with the composition of the extract.

In the present study, the cultivation, sampling, and analytical conditions were standardized across the investigated plant material, thus improving the comparability of the analyzed samples. However, the observed differences in COX inhibition cannot be attributed specifically to genotype because the experimental design did not include independent biological replication at the cultivar level and the species identity was confounded with the black-fruited group. UPLC–PDA–Q/TOF-MS profiling nevertheless demonstrated marked variation in the relative abundance of phenolic acids, flavan-3-ols and procyanidins, ellagic acid derivatives, and flavonol glycosides among the analyzed composite samples. Together with the distinct pattern of COX inhibition, these compositional differences suggest that multiple constituents of extracts may contribute to the observed activity. However, the contribution of individual metabolites or interactions among them cannot be established from the present data.

Overall, the ability of all analyzed raspberry leaf extracts to inhibit both COX-1 and COX-2 provides evidence of their in vitro activity toward an inflammation-related molecular target. Together with their diverse phenolic composition, these findings support further investigation of raspberry leaves as a source of compounds capable of modulating inflammation-related pathways. Confirmation in complementary cellular and in vivo models, as well as identification of the constituents that contribute to COX inhibition, would be required before broader conclusions regarding anti-inflammatory efficacy or potential health applications can be drawn.

Natural polyphenols have attracted considerable attention as potential modulators of carbohydrate digestion due to their ability to inhibit digestive enzymes involved in the hydrolysis of dietary carbohydrates. Among these enzymes, α-amylase and α-glucosidase catalyze key steps in the breakdown of complex carbohydrates and oligosaccharides into absorbable monosaccharides. Their inhibition can delay carbohydrate hydrolysis and is therefore considered a relevant in vitro target in the search for food-derived compounds with the potential to modulate postprandial glucose availability.

The inhibitory activity against α-amylase and α-glucosidase varied markedly among the analyzed cultivar-specific composite samples (Table 2). The Polesie sample showed the lowest IC_50_ values for both α-amylase (0.11 mg/mL) and α-glucosidase (0.09 mg/mL), indicating the strongest inhibition of both enzymes among the analyzed samples. On the contrary, higher IC_50_ values were generally observed for yellow-fruited samples. The highest overall values were recorded for Polana, with IC_50_ values of 0.80 mg/mL for α-amylase and 0.76 mg/mL for α-glucosidase. Therefore, the samples analyzed showed substantial variation in their ability to inhibit carbohydrate-hydrolyzing enzymes. Raspberry leaves are rich in several phenolic classes with reported inhibitory effects on carbohydrate-hydrolyzing enzymes, including flavan-3-ols, procyanidins, ellagitannins, flavonols, and phenolic acids. Previous phytochemical studies have consistently identified these classes as important constituents of raspberry leaves [23], providing a relevant compositional basis to investigate their potential contribution to α-amylase and α-glucosidase inhibition.

These results are consistent with those of Li et al. [29], who demonstrated that raspberry leaf extracts possess strong inhibitory activity against both α-glucosidase and α-amylase and enhance glucose consumption in 3T3-L1 cells. Using affinity ultrafiltration combined with HPLC-MS/MS and molecular coupling, the authors identified epigallocatechin gallate, procyanidin C3, rutin, cyanidin-3-rutinoside, dihydromyricetin, and isovitexin as candidate contributors to enzyme-inhibiting activity of raspberry leaf extracts. Furthermore, these compounds interacted with key catalytic amino acid residues within the active sites of both enzymes, providing mechanistic evidence supporting their inhibitory potential.

The pattern of enzyme inhibition observed in the present study did not simply parallel the total phenolic concentration of the samples analyzed. Polesie, which showed the lowest IC_50_ values for both enzymes, was characterized by a high abundance of flavan-3-ols and procyanidins. Compounds belonging to these classes have previously been reported to inhibit α-glucosidase and α-amylase, and their inhibitory potency can be influenced by structural features such as the number and position of hydroxyl groups and the degree of polymerization. The relatively high abundance of flavan-3-ols and procyanidins in the Polesie sample therefore provides a plausible compositional context for its strong enzyme-inhibitory activity; however, the contribution of individual compounds cannot be established from the present data.

Wu et al. [30] reported variation in α-glucosidase- and α-amylase-inhibitory activity among different raspberry tissues and related these differences to variation in phenolic composition, including procyanidins and ellagic acid derivatives. Together with the present results, these findings suggest that inhibition of carbohydrate-hydrolyzing enzymes by raspberry leaf extracts may be associated with the composition and relative abundance of multiple phenolic components rather than with total phenolic concentration alone.

For comparison, Bljajić et al. [31] reported no detectable α-amylase inhibition by bilberry (*Vaccinium myrtillus* L.) leaf extracts and α-glucosidase IC_50_ values of 2.53 mg/mL and 0.29 mg/mL for aqueous and ethanolic extracts, respectively. Although direct quantitative comparisons between studies should be interpreted cautiously due to differences in extraction procedures and assay conditions, these data indicate that berry leaves can represent relevant sources of constituents capable of inhibiting carbohydrate-hydrolyzing enzymes.

Overall, the observed inhibition of α-amylase and α-glucosidase indicates that raspberry leaves contain constituents capable of interacting with carbohydrate-hydrolyzing enzymes. Together with their diverse phenolic composition, these findings support further investigation of raspberry leaves as a source of compounds potentially relevant to modulation of carbohydrate digestion. However, confirmation using complementary cellular, gastrointestinal, and in vivo models would be required before conclusions can be drawn regarding effects on postprandial glycemia or broader antidiabetic potential.

### 2.3. Effects of Raspberry Leaf Preparations on Colorectal Cancer Cell Viability

The effects of raspberry leaf preparations on cell viability were evaluated in four human colorectal cancer cell lines, including LOVO and its derivative LOVO-DX (although the resistant phenotype was not independently verified under the experimental conditions used in the present study) (Figure 1), SW48 (Figure 2), and HT29 (Figure S1) and compared with their effects on the normal human colon epithelial cell line CCD 841 CoTr (Figure 2).

Raspberry leaf preparations reduced the viability of LOVO, LOVO-DX and SW48 cells over the tested concentration range, although the magnitude of the response varied between the analyzed cultivar-specific composite samples. Overall, preparations from the red-fruited samples, particularly Polana, Polesie, and Glen Ample, tended to produce greater reductions in cancer cell viability, whereas the Żółta extract generally showed a weaker effect. In LOVO cells treated with 600 μg/mL, viability was reduced to 28.4%, 29.4%, and 31.7% following treatment with Polana, Polesie, and Glen Ample preparations, respectively, compared with 47.1% for the Żółta extract (Figure 1).

The two black raspberry samples (*R. occidentalis*), Black and Nord Smelling, generally showed intermediate effects on cancer cell viability compared to the red- and yellow-fruited *R. idaeus* samples. Comparable patterns of viability reduction were observed in LOVO, LOVO-DX and SW48 cells, although the magnitude of the response varied between the analyzed samples and cell lines analyzed. LOVO-DX cells generally retained higher viability than parental LOVO cells following treatment with raspberry leaf preparations, suggesting lower sensitivity under the experimental conditions used. Nevertheless, all preparations reduced LOVO-DX cell viability over the tested concentration range. Compared to cancer cell lines, CCD 841 CoTr normal colon epithelial cells of CCD 841 CoTr generally retained higher viability, remaining above 50% throughout the tested concentration range. This pattern suggests a lower susceptibility of CCD 841 CoTr cells to the preparations under the conditions of the assay; quantitative evaluation of selectivity requires comparison of the corresponding IC_50_ values.

Unlike LOVO, LOVO-DX, and SW48 cells, HT29 cells showed a nonmonotonic and highly variable response to raspberry leaf preparations (Figure S1), including an apparent increase in PrestoBlue signal at the highest concentration tested across all samples analyzed. Because the present data do not allow this response to be unequivocally distinguished from possible interference related to the assay, the HT29 results were not used for a comparative evaluation of the potency of the extract or IC_50_ estimation and are presented separately in the Supplementary Materials (Figure S1).

A methodological limitation of cell viability experiments is that concentration-matched cell-free controls containing raspberry leaf extract and PrestoBlue were not included. Consequently, direct chemical reduction of the resazurin reagent or interference with the fluorescence signal by extract constituents cannot be excluded. This limitation is particularly relevant to the nonmonotonic response but should also be considered when interpreting the results obtained for the other cell lines. Consequently, observed responses should be interpreted as changes in cell viability measured using the PrestoBlue assay, rather than as unequivocal evidence of cytotoxicity. Future studies should include extract-only background controls and confirm representative effects using an orthogonal viability or biomass-based assay.

The reduction in colorectal cancer cell viability observed in the present study is consistent with previous reports that describe growth-inhibiting effects of raspberry leaf extracts on colorectal cancer cells. Veljković et al. [32] reported that methanolic extracts of wild *Rubus idaeus* leaves inhibited the growth of HCT-116 colorectal cancer cells, with the most active population exhibiting IC_50_ values of 162.38 μg/mL after 24 h and 95.69 μg/mL after 48 h, although considerable variability was observed among the analyzed populations. The authors also reported variations in phytochemical composition between the investigated raspberry populations, supporting a potential relationship between extract composition and effects on cancer cell growth. These findings are broadly consistent with the variation in cell viability responses observed among the raspberry leaf samples analyzed in the present study. However, a direct quantitative comparison of IC_50_ values between studies should be interpreted with caution due to differences in plant material, extraction procedures, exposure time, viability assay (MTT vs. PrestoBlue^®^), and colorectal cancer cell models.

The variation in cancer cell viability observed between the raspberry leaf samples was accompanied by differences in their phenolic composition. Samples that produced greater reductions in cell viability generally contained higher concentrations of total polyphenols and flavan-3-ols, while variation in several other phenolic groups, including phenolic acids and procyanidins, also broadly paralleled the observed differences in cancer cell viability. This pattern suggests a potential association between phenolic composition and the effects of raspberry leaf preparations on the viability of colorectal cancer cells, although the contributions of individual compounds or phenolic classes cannot be established from the present data. Raspberry leaves contain a structurally diverse mixture of catechins, procyanidins, derivatives of ellagic acids and flavonol glycosides, several of which have been associated with antiproliferative or cytotoxic effects in previous studies [33]. Therefore, the observed responses may reflect the combined effects of multiple constituents of the extracts rather than the activity of a single dominant compound.

The reduction in cancer cell viability observed for polyphenol-rich plant preparations may involve several cellular mechanisms rather than a single pathway. Catechin and epicatechin have been reported to promote ROS-associated cell death signaling in colon cancer cells, and catechin has also been reported to induce G2/M cell-cycle arrest [34]. Procyanidins, in turn, have been shown to inhibit proliferation and induce apoptosis associated with loss of mitochondrial membrane potential and caspase-3 activation in colorectal carcinoma cells, including HT-29 and LOVO cells [35]. Ellagic acid has likewise been reported to increase ROS generation, induce G2/M cell-cycle arrest, and activate apoptosis-related pathways in colon adenocarcinoma cells [36]. These findings provide a plausible mechanistic context for structurally related to those detected in raspberry leaves; however, they do not establish the mechanism responsible for the effects observed in the present study. Here, only cell viability was assessed, without measurements of intracellular ROS, mitochondrial function, apoptosis-related markers, or cell cycle distribution. Consequently, the observed reduction in colorectal cancer cell viability cannot be attributed to any specific molecular mechanism. More studies incorporating these endpoints are required to determine the pathways underlying responses to raspberry leaf preparations.

The raspberry leaf preparations also reduced the viability of LOVO-DX cells, although this cell line generally showed lower sensitivity to the preparations than the parental LOVO cells. Thus, the viability-reducing effects of the preparations were not restricted to the parental cell line. However, because resistance to doxorubicin was not experimentally verified under the conditions of the present study and the molecular mechanisms underlying the differential responses of LOVO and LOVO-DX cells were not investigated, these findings should not be interpreted as evidence that the components of raspberry leaf overcome multidrug resistance or chemoresistance. More studies incorporating appropriate chemotherapeutic controls and analysis of resistance-related mechanisms are required to address this question.

CCD 841 CoTr cells generally retained higher viability than colorectal cancer cell lines in the tested concentration range tested. The IC_50_ for CCD 841 CoTr cells was not reached for any of the seven preparations and was therefore reported as >600 μg/mL (Table S4). On the contrary, the IC_50_ values for LOVO cells ranged from 426.7 to 552.9 μg/mL, while higher values were generally observed for LOVO-DX and SW48 cells. Consequently, the calculated selectivity indices could only be expressed as lower-bound estimates, ranging from >1.09 to >1.41 for LOVO, >1.01 to >1.12 for LOVO-DX, and >1.02 to >1.35 for SW48; for the Żółta preparation, SI could not be determined for LOVO-DX or SW48 because 50% viability was not reached in either the cancer or non-malignant cell line. These results indicate a lower apparent susceptibility of CCD 841 CoTr cells, particularly relative to LOVO cells, but do not support a strong claim of selective cytotoxicity [33].

Finally, the present results further indicate that antioxidant capacity and effects on cancer cell viability should not be regarded as directly interchangeable biological properties. Although phenolic compounds can contribute to both endpoints, extracts exhibiting high antioxidant capacity do not necessarily produce the greatest reductions in cancer cell viability. Veljković et al. [32] similarly reported no positive relationship between antioxidant capacity and anticancer activity among different wild raspberry populations, despite considerable variation in their phenolic composition. These observations suggest that the effects of raspberry leaf extracts on cancer cell viability may depend on the composition and relative abundance of individual phenolic components rather than on overall antioxidant capacity alone. In the present study, potential associations between specific phenolic classes and cell viability-related parameters were further examined using exploratory correlation analysis.

### 2.4. Integrated Correlation Analysis

To explore the relationships between phenolic composition and biological responses of the analyzed raspberry leaf composite samples, Spearman’s rank correlation coefficients were calculated between the major phenolic groups and the corresponding biological activity endpoints (Figure 3). Given the small number of analyzed samples (*n* = 7), statistical significance was evaluated using exact two-sided permutation-based *p* values, followed by Benjamini–Hochberg false discovery rate (FDR) correction in all tested correlations. The analysis was considered exploratory.

The strongest FDR-significant associations were observed for flavan-3-ols, total polyphenols, and phenolic acids and derivatives. Flavan-3-ol concentration was strongly positively correlated with the antioxidant capacity measured by ABTS (ρ = 0.96, exact *p* = 0.0028, q = 0.019) and FRAP (ρ = 0.96, exact *p* = 0.0028, q = 0.019). On the contrary, the concentration of flavan-3-ol showed a perfect inverse rank correlation with cell viability at 600 μg/mL in LOVO, LOVO-DX, and SW48 cells (ρ = 1.00, exact *p* = 0.00040, q = 0.0051 for each cell line).

The total polyphenol concentration showed the same correlation pattern, including positive associations with ABTS and FRAP (ρ = 0.96, q = 0.019 for both) and inverse associations with cell viability of LOVO, LOVO-DX, and SW48 cell viability (ρ = 1.00, q = 0.0051 for each). Because the total concentration of polyphenol incorporates the major phenolic subclasses and its ranking closely paralleled that of flavan-3-ols in the present dataset, these associations should not be interpreted as independent evidence supporting the contribution of flavan-3-ols.

Phenolic acids and their derivatives also showed significant FDR associations. Their concentration was positively correlated with FRAP antioxidant capacity (ρ = 0.96, exact *p* = 0.0028, q = 0.019) and inversely correlated with LOVO, LOVO-DX, and SW48 cell viability at 600 μg/mL (ρ = −0.93, exact *p* = 0.0067, q = 0.037 for each cell line). No FDR-significant associations were observed for α-amylase or α-glucosidase IC_50_, COX-1 or COX-2, or CCD 841 CoTr viability.

In general, the exploratory correlation analysis identified flavan-3-ols, total polyphenols, and phenolic acids and derivatives as the compositional variables most consistently associated with FRAP antioxidant capacity and colorectal cancer cell viability at 600 μg/mL in the present dataset, while significant associations with ABTS were observed for flavan-3-ols and total polyphenols. No phenolic group showed an FDR-significant association with DPPH, α-amylase or α-glucosidase IC_50_, COX-1 or COX-2 inhibition, or CCD 841 CoTr viability after exact permutation-based testing. These findings should be interpreted cautiously because the analysis was based on only seven cultivar-specific composite samples, several compositional variables were mathematically or biologically interrelated, and correlation does not establish causality. Therefore, the identified associations generate hypotheses for further investigation rather than demonstrating that a particular phenolic class determines the biological activity of raspberry leaves.

## 3. Materials and Methods

### 3.1. Plant Material

Leaves were collected from seven raspberry cultivars cultivated under the same agronomic and environmental conditions at the Experimental and Educational Research Station in Samotwór, near Wrocław, Poland (51.1033° N, 16.8311° E). The plant material comprised three red-fruited *Rubus idaeus* L. cultivars (Polesie, Glen Ample, and Polana), two yellow-fruited *Rubus idaeus* L. cultivars (Poranna Rosa and Żółta), and two black-fruited *Rubus occidentalis* L. cultivars (Nord Smelling and Black). The experimental plantation was established specifically for the study using nursery-propagated plant material. The provenance and identity were documented by the plant passports supplied with the planting material and by the corresponding plantation records maintained at the Experimental and Educational Research Station. No herbarium voucher specimens were deposited because the plant material was obtained from established cultivated plants with documented nursery provenance and plant passports.

Healthy, fully expanded leaves were harvested during the fruiting stage on 30 June 2024. For each cultivar, leaves were collected from the upper, middle, and lower parts of five individual shrubs. Immediately after collection, the leaves obtained from the five shrubs were pooled to form a single composite sample representing each cultivar. Thus, a pooled composite leaf sample was obtained for each of the seven cultivars and subsequently used for chemical and biological analyses.

Fresh leaves were spread in a thin layer on Whatman filter paper (Whatman^®^, Maidstone, UK) and air-dried for seven days in the dark at 22 °C with natural ventilation until constant weight was reached. To ensure uniform drying, the leaves were gently turned every two days. After drying, the material was vacuum-packed and stored at −20 °C until further analysis. Before extraction, each composite sample was ground into a fine homogeneous powder using a laboratory grinder.

### 3.2. Preparation of Methanolic Extracts

The dried raspberry leaf samples were ground to a fine powder using a laboratory mill (IKA A11; Staufen, Germany). For each cultivar-specific composite sample, two separate 100 mg portions of the homogenized powder were weighed and extracted independently. Each portion was extracted with 1 mL of 80% methanol acidified with 1% HCl (*v*/*v*). The extraction was carried out for 24 h at 4 ° C. The samples were sonicated twice for 20 min in an ultrasonic bath (300 W, 40 kHz; Sonic 6D, Polsonic, Warsaw, Poland), immediately after adding the solvent and again after 24 h of extraction. During the extraction period, the samples were periodically mixed to facilitate solvent penetration.

After extraction, the samples were centrifuged at 19,000× *g* for 10 min at 4 ° C. The supernatants were filtered once through 0.20 μm hydrophilic PTFE membrane filters (Millex Samplicity™, Merck, Darmstadt, Germany). The two independently prepared extracts obtained from each cultivar-specific composite sample were subsequently used for UPLC–PDA–Q/TOF-MS analysis and antioxidant-capacity assays. Separate larger-scale extracts were prepared and purified as described below for enzyme-inhibition, COX-inhibition, and cell viability assays.

For the preparation of the fractions of raspberry leaves used in the biological assays, 100 g of dried leaves from each cultivar-specific composite sample were extracted with 1.6 L of 80% methanol acidified with 1% HCl (*v*/*v*), using the same extraction conditions as described above. The extraction procedure consisted of sonication for 20 min, storage at 4 ° C for 24 h, a second 20 min sonication step, and centrifugation. Subsequently, the methanol was removed under reduced pressure using a rotary evaporator, and the resulting concentrated extract was subjected to column chromatography on Amberlite resin. The column was washed with distilled water to reduce the content of water-soluble matrix constituents, including sugars and organic acids. The effluent was monitored refractometrically (°Brix), and washing was continued until a value of 0 °Brix was reached. The fraction retained in the resin was subsequently eluted with 96% (*v*/*v*) ethanol. The collected ethanolic eluate was concentrated under reduced pressure, frozen, and lyophilized. Approximately 0.30 g of purified lyophilized preparation per 100 g of dried leaf material. The resulting preparations were used in biological assays.

### 3.3. UPLC–PDA–Q/TOF-MS Analysis of Phenolic Compounds

Phenolic compounds were analyzed using ultra-performance liquid chromatography coupled with photodiode array detection and quadrupole time-of-flight mass spectrometry (UPLC–PDA–Q/TOF-MS), according to a previously described method with minor modifications [37]. Extracts were analyzed using an ACQUITY Ultra Performance LC system equipped with a binary solvent manager and photodiode array detector (Waters Corp., Milford, MA, USA) coupled to a G2 Q-ToF mass spectrometer (Waters, Manchester, UK) fitted with an electrospray ionization (ESI) source operating in negative ion mode.

Chromatographic separation was performed on an ACQUITY UPLC BEH C18 column (100 × 2.1 mm, 1.7 µm; Waters Corp., Milford, MA, USA) maintained at 30 °C. The mobile phase consisted of solvent A (0.1% formic acid in water, *v*/*v*) and solvent B (acetonitrile). The injection volume was 10 µL, and the flow rate was maintained at 0.42 mL/min. The chromatographic gradient was identical to that described previously.

Mass spectra were acquired over the *m*/*z* range of 100–2500 using data-dependent acquisition (DDA). Leucine enkephalin (500 pg/µL) was continuously infused as the lock-mass reference compound via the LockSpray interface using the [M–H]^−^ ion at *m*/*z* 554.2615 for real-time mass correction. The optimized operating conditions were as follows: capillary voltage, 2.5 kV; cone voltage, 30 V; source temperature, 100 °C; desolvation temperature, 300 °C; and nitrogen desolvation gas flow, 300 L/h. Collision-induced dissociation experiments were performed using argon as the collision gas with collision energy ramped from 0.3 to 2.0 V.

Phenolic compounds were identified based on their retention times, UV–Vis spectra, molecular masses, and characteristic MS/MS fragmentation patterns. Data acquisition and processing were performed using MassLynx 4.0 with the ChromaLynx Application Manager (Waters, Manchester, UK).

Chromatograms were monitored at 280 nm (flavan-3-ols and related compounds), 320 nm (phenolic acids), and 360 nm (flavonols), and the PDA spectra were recorded over the wavelength range of 200–600 nm.

Quantification was performed using external calibration curves prepared from commercially available reference materials. Compounds for which the corresponding authentic standard was available were quantified directly using the calibration curve of that standard. For compounds without an authentic reference standard, a semiquantitative determination was performed using the calibration curve of a structurally related reference compound, and the resulting concentrations were expressed as equivalents of the respective standard. Quinic acid and chlorogenic acid derivatives were quantified as 3-caffeoylquinic acid equivalents. Gallic acid and its derivatives were expressed as gallic acid equivalents, while ellagic acid derivatives were quantified as ellagic acid equivalents. Caffeic acid derivatives were quantified using caffeic acid as the standard. Flavan-3-ols, A-type procyanidins, and B-type procyanidins were quantified using (+)-catechin, procyanidin A2, and procyanidin B2 as standards, respectively. Quercetin, kaempferol, and apigenin derivatives were quantified using quercetin 3-O-glucoside, kaempferol 3-*O*-glucoside, and apigenin 7-*O*-glucoside as standards, respectively. Accordingly, values obtained using surrogate standards should be interpreted as semi-quantitative estimates rather than absolute concentrations of the individual compounds. The reference standards used for quantitative calibration should not be conflated with the authentic standards used for compound identification; only gallic acid, caffeic acid, neochlorogenic acid, and (+)-catechin were confirmed by comparison with the authentic standards and were therefore assigned identification level 1. The results are expressed as mg/kg of dry weight (DW). For each cultivar, two extracts were independently prepared from the corresponding pooled composite sample, as described in Section 3.2. Each extract was analyzed using three replicate UPLC injections to assess instrumental repeatability. The replicate injections were treated as instrumental replicates and not as independent biological observations.

### 3.4. Determination of Antioxidant Capacity and Enzyme-Inhibitory Activities

The antioxidant capacity of raspberry leaf extracts was determined using the ABTS, DPPH, and ferric-reducing antioxidant power (FRAP) assays, according to the procedures described by Chandran et al. [38]. All measurements were performed using a Synergy H1 microplate reader (BioTek Instruments, Winooski, VT, USA). The antioxidant capacity was expressed as μmol Trolox equivalents (TE) per 100 g dry weight (DW).

The purified lyophilized raspberry leaf preparations were initially dissolved in 80% methanol acidified with 1% HCl (*v*/*v*) to obtain stock solutions at 40 mg/mL. For the α-glucosidase and α-amylase assays, the appropriate aliquots of these stock solutions were further diluted with the corresponding assay buffers (0.1 M potassium phosphate buffer, pH 6.9, for α-glucosidase and 0.1 M phosphate buffer containing 6 mM CaCl_2_, pH 6.9, for α-amylase) to obtain working solutions that yield final preparation concentrations of 0.25, 0.50, and 1.00 mg/mL in the reaction mixture. These concentrations corresponded to final methanol concentrations of 0.50%, 1.00%, and 2.00% (*v*/*v*), respectively.

The inhibitory activity of the raspberry leaf preparations against α-glucosidase and α-amylase was evaluated at concentrations of 0.25, 0.50, and 1.00 mg/mL according to the procedure described previously [38], with modifications as specified below. For the α-glucosidase assay, intestinal acetone powder of rat origin was used as the enzyme source and p-nitrophenyl-α-D-glucopyranoside (pNPG) as the substrate. Intestinal acetone powder of rat origin, p-nitrophenyl-α-D-glucopyranoside (pNPG), porcine pancreatic α-amylase (type VI-B), potato starch, and acarbose were obtained from Sigma-Aldrich (currently Merck, Darmstadt, Germany). Enzyme preparation was obtained by suspending 0.5 g of intestinal acetone powder in 10 mL of 0.9% NaCl, followed by sonication and centrifugation; the resulting supernatant was diluted twice with 0.1 M potassium phosphate buffer (pH 6.9). Subsequently, 50 μL of the tested preparation was mixed with 50 μL of the enzyme solution and preincubated for 10 min. The reaction was initiated by adding 50 μL of 5 mM pNPG and continued for 20 min at 37 °C. The reaction was terminated by adding 100 μL of 0.1 M Na_2_CO_3_, and absorbance was measured at 405 nm.

α-Amylase-inhibitory activity was determined using porcine pancreatic α-amylase (type VI-B) and potato starch as the substrate. The reaction mixture contained 40 μL of 0.2% (*w*/*v*) potato starch solution, 20 μL of the tested preparation, and 20 μL of porcine pancreatic α-amylase (type VI-B) solution prepared at 0.1 mg/mL in 0.1 M phosphate buffer containing 6 mM CaCl_2_ (pH 6.9). After incubation for 10 min at 37 °C, the reaction was terminated by adding 80 μL of 0.4 M HCl, followed by 100 μL of 5 mM I_2_ in 5 mM KI. Absorbance was measured at 600 nm. Appropriate sample blanks and enzyme controls were included in both assays to correct for background absorbance. Enzyme inhibition was calculated relative to the corresponding enzyme control after blank correction and expressed as percentage inhibition. Acarbose at 0.05 and 0.20 mg/mL was used as a positive reference inhibitor.

The percentage inhibition values obtained at preparation concentrations of 0.25, 0.50, and 1.00 mg/mL were fitted by linear regression according to the equation y = ax + b, where y represents enzyme inhibition (%) and x represents preparation concentration (mg/mL). IC_50_, defined as the concentration required to inhibit enzyme activity by 50%, was estimated from the fitted equation as IC_50_ = (50 − b)/a.

The inhibitory activities of COX-1 and COX-2 were determined spectrophotometrically according to a previously described method [38], with modifications. Ovine COX-1 (≥40,000 units/mg protein), recombinant human COX-2 expressed in baculovirus-infected Sf21 cells (≥8000 units/mg protein), hematin, N,N,N′,N′-tetramethyl-p-phenylenediamine (TMPD), arachidonic acid, and ibuprofen were obtained from Sigma-Aldrich (currently Merck, Darmstadt, Germany). The assay was performed in 0.1 M Tris-HCl buffer (pH 8.0) at 25 °C. The COX working solution was prepared by diluting the enzyme stock (1 mg/mL) with Tris-HCl buffer to 0.033 mg/mL. The tested preparations were added from the 40 mg/mL stock solutions described above, yielding a final preparation concentration of 1.00 mg/mL and a final methanol concentration of 2.00% (*v*/*v*) in the reaction mixture. The final reaction mixture (200 μL) contained 5 μL of the tested preparation, 90 μL of the working solution, 10 μL of hematin solution 8 μL of TMPD solution, and 87 μL of arachidonic acid solution. The final concentrations of hematin, TMPD, and arachidonic acid in the reaction mixture were approximately 0.51 μM, 0.24 mM, and 0.76 mM, respectively. After addition of the preparation, COX and hematin, the plate was mixed for 30 s and incubated for 2.5 min. TMPD and arachidonic acid were then added to initiate the reaction, followed by mixing for 5 s. The absorbance was measured at 611 nm immediately after the initiation of the reaction and after 1 min. DMSO-containing wells in which COX was replaced with buffer were used as background controls, whereas DMSO-containing wells with COX served as enzyme controls. COX inhibition was calculated as [(ΔAcontrol − ΔAsample)/ΔAcontrol] × 100, where ΔAcontrol and ΔAsample represent the change in absorbance during the 1-min reaction period in the enzyme control and sample-containing wells, respectively. The inhibition values for COX-1 and COX-2 reported in Table 2 were determined at a final raspberry leaf preparation concentration of 1.00 mg/mL. Ibuprofen at a final concentration of 1.00 mg/mL was used as the positive reference inhibitor.

For each cultivar, the two independently prepared extracts obtained from the corresponding pooled composite leaf sample were analyzed separately. For each extract and assay condition, measurements were performed in four replicate wells, which were treated as technical assay replicates and not as independent biological observations.

### 3.5. Cell Culture and In Vitro Assays

#### 3.5.1. Cell Culture

The human colorectal adenocarcinoma cell lines LOVO, LOVO-DX, SW48, and HT29, and the normal human colon epithelial cell line CCD 841 CoTr, were obtained from the American Type Culture Collection (ATCC, Manassas, VA, USA). Cells were cultured under standard conditions at 37 °C in a humidified atmosphere containing 5% CO_2_ in the appropriate complete growth medium supplemented with 10% fetal bovine serum (FBS), 2 mM L-glutamine, and gentamicin. Cells were routinely passaged twice a week using TrypLE™ Express solution (Thermo Fisher Scientific, Waltham, MA, USA). Biological assays were performed using purified lyophilized preparations obtained from the crude methanolic extracts by Amberlite column purification.

#### 3.5.2. Cell Viability Assay

The effects of raspberry leaf preparations on cell viability were evaluated using PrestoBlue^®^ Cell Viability Reagent (Thermo Fisher Scientific, Waltham, MA, USA), which is based on the reduction of resazurin by metabolically active cells. Cells were seeded into 96-well microplates and allowed to attach overnight under standard culture conditions. Subsequently, the culture medium was replaced with fresh medium containing raspberry leaf preparations at concentrations ranging from 50 to 600 μg/mL.

For the cell-viability experiments, the purified lyophilized raspberry leaf preparations were initially dissolved in DMSO to obtain stock solutions at 200 mg/mL. Working solutions at 20 mg/mL were subsequently prepared by a 10-fold dilution of the stock solutions with the appropriate cell-culture medium. Treatment solutions containing 50, 100, 200, 300, 400, or 600 μg/mL of the lyophilized preparation were prepared in culture medium. The corresponding final DMSO concentrations were 0.025%, 0.05%, 0.10%, 0.15%, 0.20%, and 0.30% (*v*/*v*), respectively. A vehicle control containing 0.30% (*v*/*v*) DMSO, corresponding to the highest DMSO concentration present in the treatment solutions, was included. All solutions were prepared aseptically under conditions appropriate for cell-culture experiments and were not sterile-filtered.

For each cultivar, concentration series were prepared from each of the two independently prepared extracts obtained from the corresponding pooled composite leaf sample. Each extract concentration was tested in four replicate wells, which were treated as technical assay replicates.

After 72 h of incubation, 10 μL of PrestoBlue reagent was added to each well containing 90 μL of culture medium and incubated for 1 h at 37 °C. Fluorescence was measured using a Varioskan LUX multimode microplate reader (Thermo Fisher Scientific, Waltham, MA, USA) at excitation and emission wavelengths of 560 and 590 nm, respectively. Cell viability was expressed as the percentage of viable cells relative to the untreated control.

IC_50_ values were estimated by linear interpolation between the two adjacent concentrations tested that included 50% cell viability, using the mean viability values obtained from the two independently prepared extracts. When cell viability remained above 50% at the maximum concentration tested (600 μg/mL), the IC_50_ was reported as >600 μg/mL, and no extrapolation was carried out beyond the range of concentrations tested experimentally. Selectivity indices (SI) were calculated as the ratio of the IC_50_ value for the non-malignant CCD 841 CoTr cells to that for the respective colorectal cancer cell line. Because the IC_50_ for CCD 841 CoTr cells exceeded 600 μg/mL for all preparations, the corresponding SI values were reported as estimates (>). When the IC_50_ exceeded 600 μg/mL for both cancer and non-malignant cell lines, SI was considered indeterminate within the concentration range.

### 3.6. Statistical Analysis

Statistical analyses and data processing were performed using Statistica 13.1 software (TIBCO Software Inc., Palo Alto, CA, USA), GraphPad Prism version 6.05 (GraphPad Software, San Diego, CA, USA) and Python version 3.13.5. Exact permutation-based *p*-values for Spearman rank correlations were calculated using Python, and multiple test correction was performed using the Benjamini–Hochberg false discovery rate procedure.

For each cultivar, the plant material collected from five shrubs was pooled to form a single composite sample. Two extracts were independently prepared from each composite sample, whereas replicate UPLC injections and replicate wells in the microplate-based assays represented instrumental and technical replicates, respectively. For UPLC–PDA–Q/TOF-MS analysis, each of the two independently prepared extracts was analyzed using three replicate injections. For the antioxidant and enzyme inhibition assays, each extract was analyzed in four technical replicate wells per assay condition. Because the experimental design included a pooled composite biological sample per cultivar, extraction, instrumental, and technical replicates were not treated as independent biological observations for cultivar-level statistical inference. Consequently, comparisons among the seven cultivar-specific composite samples were descriptive, and no inferential statistical tests were used to establish differences between cultivars.

For cell viability assays, concentration series were prepared separately from each of the two independently prepared extracts, and each concentration was analyzed in four technical replicate wells per extract. Cell viability was expressed relative to the corresponding untreated control and summarized descriptively.

The associations between phenolic composition and biological activity endpoints were explored using Spearman’s rank correlation coefficients (ρ) across the seven cultivar-specific composite samples (*n* = 7). The compositional variables included the major phenolic groups and the total concentration of polyphenols. Biological endpoints included antioxidant capacity determined by the ABTS, DPPH, and FRAP assays; IC_50_ values for α-amylase and α-glucosidase inhibition; COX-1 and COX-2 inhibition; and cell viability of LOVO, LOVO-DX, SW48, and CCD 841 CoTr cells at the highest tested extract concentration (600 μg/mL). HT29 cells were excluded from the correlation analysis because their nonmonotonic and highly variable concentration–response pattern precluded reliable comparative interpretation. Given the small number of observations, the statistical significance of Spearman’s correlations was evaluated using exact two-sided permutation-based *p*-values. To account for multiple tests, the *p*-values of the 77 correlations (7 compositional variables × 11 biological endpoints) were adjusted using the Benjamini–Hochberg false discovery rate (FDR) procedure, with q < 0.05 considered statistically significant. The correlation coefficients were visualized as a heat map. Due to the limited sample size and interdependence of some compositional variables, the correlation analysis was considered exploratory and the observed associations were not interpreted as evidence of causal relationships.

## 4. Conclusions

The present study demonstrated that raspberry leaves are a valuable source of polyphenolic compounds with various biological properties, although their composition and biological responses varied considerably among the analyzed cultivar-specific composite samples. The observed patterns differed depending on the biological endpoint and should not be attributed independently to the cultivar/genotype, the color of the fruit or species, as these factors could not be separated within the present experimental design.

Flavan-3-ols and procyanidins represented the major components of the phenolic fraction, with an A-type procyanidin trimer and several B-type procyanidin dimers occurring among the most abundant individual constituents, while quercetin *O*-pentoside isomer 2 was a prominent flavonol in all analyzed samples. (+)-Catechin, confirmed using an authentic standard, was also detected in all seven samples. Exploratory correlation analysis using exact permutation-based significance testing followed by FDR correction identified flavan-3-ols and total polyphenol concentration as compositional variables most consistently associated with ABTS and FRAP antioxidant capacity and with reduced cell viability of LOVO, LOVO-DX and SW48 cell viability at 600 μg/mL. Phenolic acids and their derivatives were also positively associated with FRAP antioxidant capacity and inversely associated with the viability of these three cancer cell lines. On the contrary, no phenolic group showed a significant association with α-amylase or α-glucosidase IC_50_ values, inhibition of COX-1 or COX-2, or CCD 841 CoTr cell viability. In particular, the strongest inhibition of COX-1 and COX-2 was observed for the Poranna Rosa sample, indicating that COX inhibition activity did not simply parallel total phenolic concentration or the abundance of flavan-3-ols and procyanidins.

These findings highlight raspberry leaves as a promising underutilized plant material with potential applications as a source of natural bioactive ingredients for functional foods, nutraceuticals, and other value-added products. At the same time, the present findings should be regarded as a basis for further investigation rather than as evidence of the superiority of particular cultivars or groups of fruit color. Independent biological replication across a broader set of cultivars within both *R. idaeus* and *R. occidentalis* will be necessary to disentangle the effects of cultivar/genotype, species, and fruit color. Further studies should also confirm the observed biological responses using complementary experimental models, investigate the contributions and mechanisms of individual phenolic constituents, and establish the reproducibility and standardization of raspberry leaf preparations before their potential application as functional ingredients can be assessed.

## Figures and Tables

**Figure 1:**
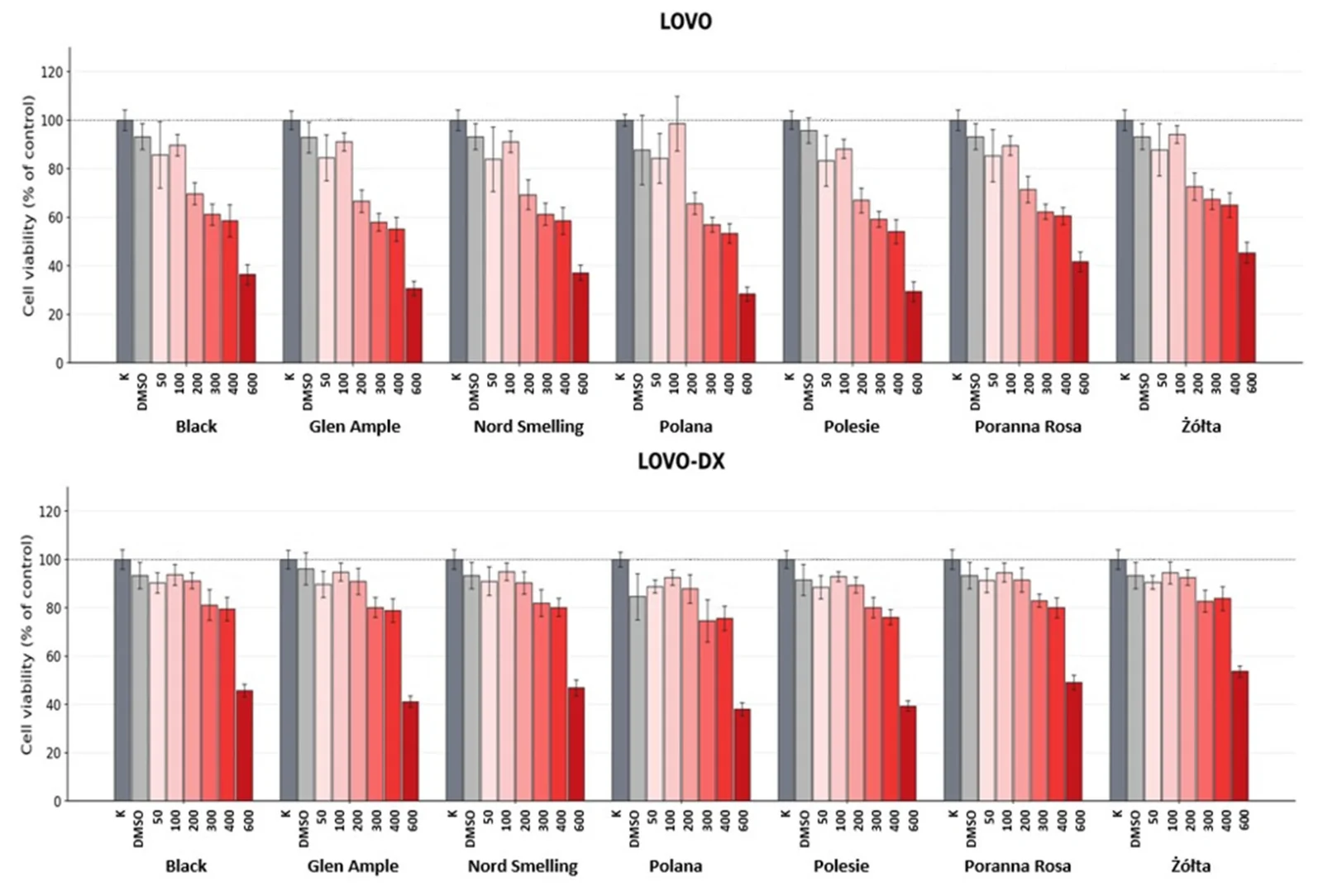
Effects of raspberry leaf preparations on the viability of LOVO and LOVO-DX colorectal cancer cells after 72 h of treatment. Cells were exposed to extract concentrations ranging from 50 to 600 μg/mL. For each cultivar, concentration series were prepared separately from two independently prepared preparations obtained from the corresponding pooled composite leaf sample. Each concentration was analyzed in four technical replicate wells for each extract. Values are presented as mean ± SD calculated from the two extraction-level mean values. DMSO, solvent control.

**Figure 2:**
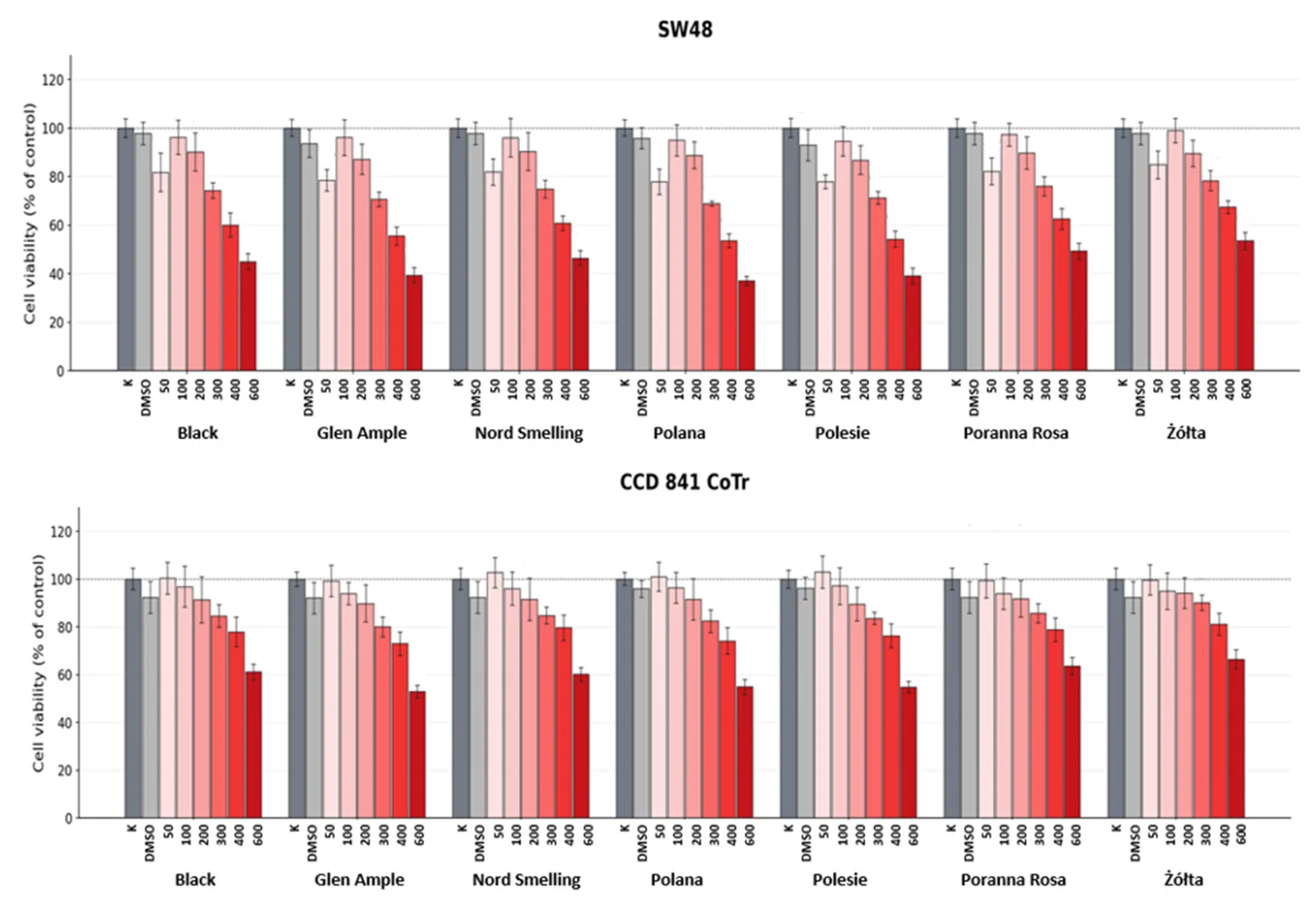
Effects of raspberry leaf preparations from seven cultivars on the viability of SW48 human colorectal adenocarcinoma cells and CCD 841 CoTr normal human colon epithelial cells after 72 h of treatment. Cell viability was determined using the PrestoBlue^®^ assay and expressed as the percentage of viable cells relative to untreated controls (100%). Each panel includes the untreated control (K), DMSO solvent control, and raspberry leaf preparations concentrations ranging from 50 to 600 µg/mL. For each cultivar, concentration series were prepared separately from two independently prepared preparations obtained from the corresponding pooled composite leaf sample. Each concentration was analyzed in four technical replicate wells for each extract. Values are presented as mean ± SD calculated from the two extraction-level mean values.

**Figure 3:**
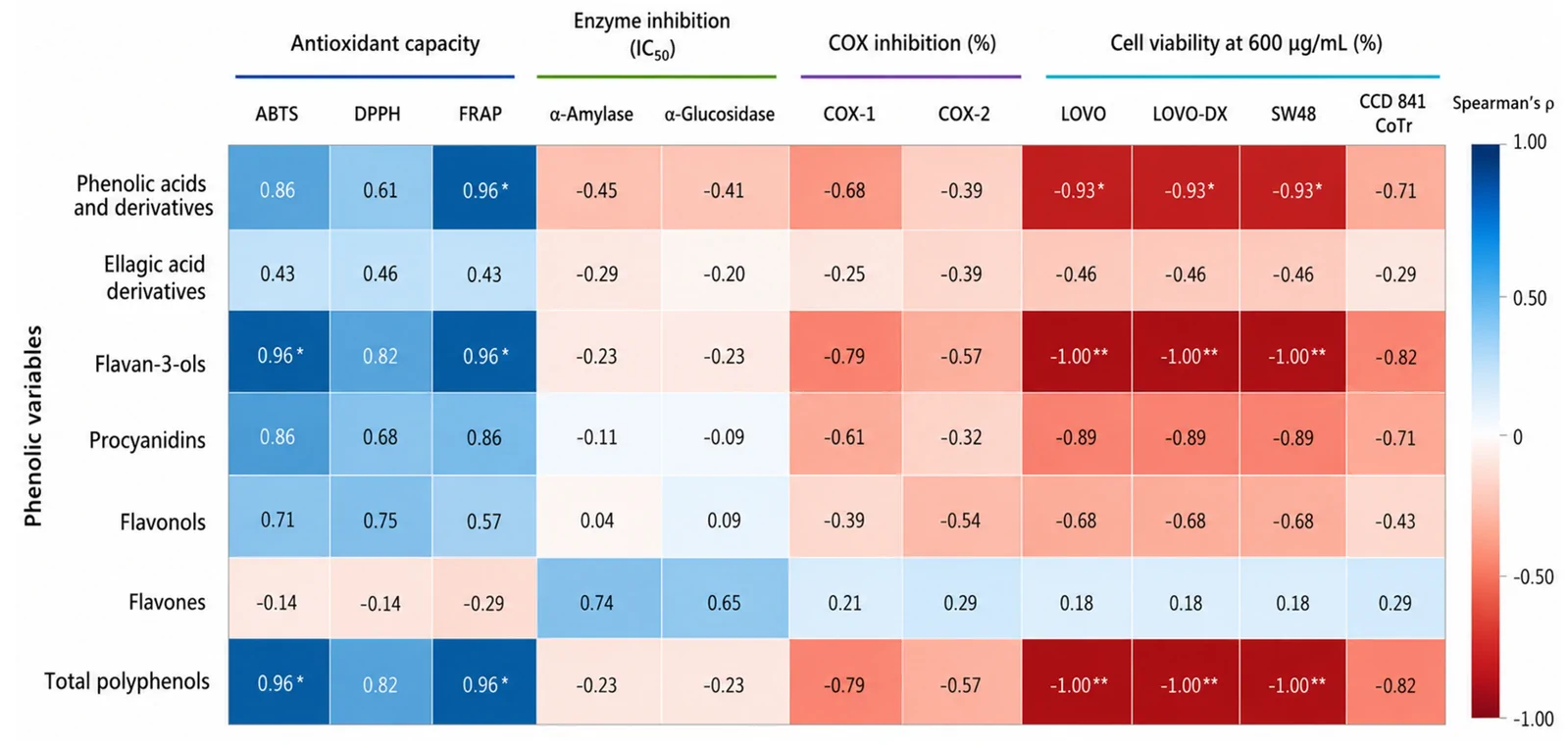
Spearman correlation heatmap illustrating associations between major phenolic groups and biological activity endpoints across the seven analyzed cultivar-specific raspberry leaf composite samples (*n* = 7). Cell viability corresponds to the response measured at the highest tested extract concentration (600 μg/mL). Lower α-amylase and α-glucosidase IC_50_ values and lower cell viability indicate stronger effects, whereas higher COX inhibition values indicate greater COX-inhibitory activity. Values represent Spearman’s rank correlation coefficients (ρ). Statistical significance was evaluated using exact two-sided permutation-based *p*-values followed by Benjamini–Hochberg false discovery rate correction across all 77 tested correlations. * q < 0.05; ** q < 0.01. Exact *p*-values and FDR-adjusted q-values for all correlations are provided in Table S3. The analysis was considered exploratory owing to the limited number of samples.

**Table 1:** Contents of identified and tentatively identified phenolic compounds in raspberry leaves (mg/kg DW).

Tentative Identification	Red			Yellow		Black	
	Glen Ample	Polesie	Polana	Żółta	Poranna Rosa	Nord Smelling	Black
Phenolic acids and derivatives							
Quinic acid	nd	nd	nd	114.16 ± 8.09	125.01 ± 17.62	121.51 ± 0.69	121.53 ± 10.32
Gallic acid *O*-hexoside	110.06 ± 0.65	464.72 ± 22.61	447.46 ± 52.63	269.7 ± 11.27	291.38 ± 17.09	285.35 ± 1.48	288.75 ± 8.60
Gallic acid	nd	nd	nd	99.28 ± 5.60	111.07 ± 14.86	109.01 ± 2.18	110.74 ± 11.00
Neochlorogenic acid	455.80 ± 4.97	462.09 ± 31.14	475.85 ± 37.03	167.59 ± 7.12	184.18 ± 10.10	275.38 ± 4.65	290.14 ± 8.73
Ellagic acid *O*-pentoside	243.64 ± 3.76	235.63 ± 8.60	256.42 ± 17.73	71.55 ± 4.84	79.08 ± 14.61	nd	nd
Caffeic acid	250.38 ± 3.49	262.55 ± 25.26	252.47 ± 19.85	176.15 ± 7.28	202.18 ± 0.67	173.74 ± 40	180.45 ± 5.75
Caffeic acid *O*-hexoside isomer 1	nd	nd	nd	170.39 ± 3.60	188.47 ± 20.69	172.53 ± 2.26	170.75 ± 6.26
Caffeic acid *O*-hexoside isomer 2	947.49 ± 3.69	999.45 ± 75.17	952.42 ± 104.20	193.37 ± 3.30	212.22 ± 24.04	327.72 ± 2.59	334.26 ± 39.03
Chlorogenic acid	627.62 ± 4.02	656.88 ± 26.84	625.92 ± 36.38	492.78 ± 5.97	541.57 ± 52.16	499.56 ± 4.13	533.91 ± 24.55
Caffeic acid *O*-hexoside isomer 3	309.21 ± 1.89	321.96 ± 28.20	321.45 ± 10.93	114.75 ± 1.89	121.15 ± 6.41	nd	nd
Caffeic acid *O*-hexoside isomer 4	nd	nd	nd	55.13 ± 0.98	58.23 ± 5.65	1652.72 ± 4.26	1717.92 ± 57.51
Caffeoylthreonic acid isomer	1040.78 ± 4.42	1087.06 ± 12.31	1081.20 ± 74.89	1706.08 ± 5.61	1883.7 ± 122.01	nd	nd
Caffeoylquinic acid isomer	1129.15 ± 3.58	1219.02 ± 65.35	1154.41 ± 92.95	746.39 ± 4.65	815.14 ± 30.96	858.27 ± 4.19	912.24 ± 34.39
Ellagic acid acetyl pentoside isomer 1	665.15 ± 7.52	695.69 ± 61.27	670.77 ± 59.31	346.69 ± 3.66	398.21 ± 7.82	475.12 ± 9.24	466.38 ± 38.63
Ellagic acid acetyl pentoside isomer 2	3145.31 ± 18.74	3138.05 ± 221.09	3291.23 ± 93.81	2096.23 ± 2.85	2242.65 ± 47.55	3784.19 ± 16.42	3896.91 ± 352.35
p-Coumaroyl-caffeoylquinic acid isomer	6.00 ± 0.23	6.30 ± 0.10	5.81 ± 0.52	2.97 ± 0.04	3.09 ± 0.13	4.15 ± 0.20	4.10 ± 0.06
Caffeoylglucose derivative	4.18 ± 0.23	4.13 ± 0.03	4.39 ± 0.45	1.68 ± 0.03	1.86 ± 0.17	2.85 ± 0.08	3.00 ± 0.16
Dicaffeoylquinic acid isomer	355.70 ± 18.36	372.38 ± 52.12	359.97 ± 1.77	208.89 ± 2.34	230.19 ± 14.91	290.45 ± 16.57	300.37 ± 13.65
Sum	9290.47 ± 483.1	9925.91 ± 406.96	9899.77 ± 623.69	7033.78 ± 330.59	7689.38 ± 445.98	9032.55 ± 325.17	9331.45 ± 513.23
Flavan-3-ols and procyanidins							
Epicatechin	1006.26 ± 8.56	1013.97 ± 69.64	1053.49 ± 113.56	nd	nd	nd	nd
B-type Procyanidin dimer isomer 1	3622.47 ± 16.89	3612.33 ± 329.34	3851.11 ± 197.25	1814.67 ± 14.04	2057.10 ± 140.47	1763.62 ± 12.87	1825.13 ± 194.13
B-type Procyanidin dimer isomer 2	7552.91 ± 44.14	7610.04 ± 210.04	7792.15 ± 499.49	3518.09 ± 28.09	3932.28 ± 384.31	4442.40 ± 35.30	4580.52 ± 136.92
B-type Procyanidin dimer isomer 3	5377.26 ± 35.44	5545.86 ± 614.48	5547.23 ± 602.22	10,596.57 ± 35.12	11,115.33 ± 674.48	9001.88 ± 28.41	8973.79 ± 659.13
B-type Procyanidin dimer isomer 4	1630.34 ± 22.11	1718.15 ± 11.28	1623.58 ± 106.17	332.04 ± 6.96	357.13 ± 19.00	575.59 ± 11.74	604.88 ± 61.98
B-type Procyanidin trimer	1275.78 ± 16.76	1246.01 ± 48.58	1374.96 ± 65.87	649.55 ± 13.81	692 ± 64.67	nd	nd
A-type Procyanidin trimer	11,854.37 ± 67.99	12,230.45 ± 1000.57	12,225.11 ± 860.30	7585.65 ± 95.24	7962.17 ± 500.74	9553.4 ± 118.51	9717.53 ± 372.55
Catechin	3204.34 ± 12.93	3189.58 ± 152.53	3377.51 ± 81.60	1162.97 ± 8.02	1280.60 ± 55.20	2033.18 ± 11.67	2021.32 ± 132.36
Catechin gallate	5625.18 ± 78.73	5800.01 ± 256.88	5666.51 ± 43.98	2069.56 ± 34.59	2339.64 ± 134.13	2935.75 ± 54.66	3038.17 ± 179.02
Sum	41,148.91 ± 1604.81	41,966.4 ± 2559.95	42,511.65 ± 1870.51	27,729.1 ± 1830.12	29,736.25 ± 1486.81	30,305.82 ± 1727.43	30,761.34 ± 1322.74
Flavonols							
Kaempferol glycoside derivative	885.18 ± 3.63	920.00 ± 65.70	868.00 ± 46.75	409.87 ± 3.93	428.95 ± 17.71	722.55 ± 5.39	718.69 ± 61.06
Quercetin *O*-rutinoside	nd	nd	nd	nd	nd	926.97 ± 25.18	934.76 ± 121.06
Kaempferol pentosyl-hexuronide	838.58 ± 10.21	838.21 ± 87.29	860.78 ± 75.71	813.42 ± 5.66	874.32 ± 72.44	751.53 ± 8.77	773.80 ± 38.79
Quercetin *O*-pentoside isomer 1	848.17 ± 5.04	897.60 ± 57.64	829.82 ± 32.85	nd	nd	nd	nd
Quercetin *O*-pentoside isomer 2	8608.60 ± 14.47	8547.77 ± 667.39	8682.60 ± 994.64	8627.40 ± 6.45	9221.27 ± 800.77	9325.26 ± 13.13	9222.22 ± 646.41
Quercetin HMG-hexoside	654.64 ± 7.48	680.39 ± 11.21	681.16 ± 47.76	622.29 ± 4.81	654.36 ± 57.15	681.02 ± 9.68	705.40 ± 86.10
Quercetin *O*-pentoside isomer 3	1411.73 ± 15.91	1417.94 ± 133.75	1490.04 ± 134.57	850.32 ± 3.97	898.51 ± 58.71	1013.93 ± 13.37	1062.44 ± 67.78
Kaempferol *O*-hexuronide	1497.02 ± 16.69	1473.58 ± 26.97	1526.90 ± 140.86	1795.57 ± 3.80	1930.75 ± 144.93	1835.56 ± 14.20	1798.34 ± 121.63
Kaempferol *O*-pentoside	1219.03 ± 30.36	1231.42 ± 144.41	1276.24 ± 73.35	574.19 ± 3.56	653.39 ± 32.33	824.63 ± 25.35	859.24 ± 105.09
Sum	15,962.95 ± 1021.63	16,006.91 ± 768.33	16,215.54 ± 599.97	13,693.06 ± 739.43	14,661.55 ± 879.69	16,081.45 ± 723.67	16,074.89 ± 819.82
Flavones							
Apigenin *O*-pentoside	875.93 ± 10.08	861.68 ± 21.70	920.61 ± 54.08	951.30 ± 5.51	887.32 ± 30.60	810.84 ± 7.05	297.28 ± 40.19
TOTAL:	67,278.26 ± 3969.42	68,760.9 ± 2887.96	69,547.57 ± 3407.83	49,407.24 ± 3310.29	52,974.5 ± 1854.11	56,230.66 ± 3092.69	56,464.96 ± 3444.36

Values are presented as mean ± standard deviation (SD) based on two independently prepared extracts obtained from a single pooled composite leaf sample for each cultivar. Each extract was analyzed using three replicate UPLC injections, which were treated as instrumental replicates. The mean of the three injections was calculated for each extract, and the reported mean ± SD was subsequently calculated from the two extraction-level means. Thus, the two extracts represent extraction replicates rather than independent biological replicates. Accordingly, no inferential statistical comparisons among cultivar-specific composite samples were performed. Abbreviation: nd, not detected.

**Table 2:** Antioxidant capacity and enzyme-inhibitory potential in the different raspberry leaves.

Raspberry Color	Cultivar	ABTS	DPPH	FRAP	α-Amylase	α-Glucosidase	COX-1	COX-2
		μmol Trolox Equivalents (TE)/100 g DW			IC_50_ = (mg/mL)		Inhibition (%)	
Red raspberry	Glen Ample	20.03 ± 1.62	20.44 ± 1.21	25.27 ± 1.85	0.13 ± 0.01	0.10 ± 0.01	61.87 ± 1.8	52.31 ± 4.22
	Polana	20.80 ± 1.72	20.84 ± 1.33	27.97 ± 2.01	0.80 ± 0.06	0.76 ± 0.04	63.16 ± 1.9	55.43 ± 4.12
	Polesie	20.45 ± 1.60	18.54 ± 1.14	29.58 ± 2.10	0.11 ± 0.01	0.09 ± 0.00	62.18 ± 1.7	55.87 ± 5.05
Yellow raspberry	Poranna Rosa	18.47 ± 1.52	18.00 ± 1.00	21.47 ± 1.54	0.61 ± 0.04	0.51 ± 0.00	85.53 ± 2.3	66.11 ± 2.28
	Żółta	18.24 ± 1.51	16.91 ± 1.01	17.80 ± 1.37	0.63 ± 0.05	0.51 ± 0.00	83.25 ± 2.1	60.48 ± 2.74
Black raspberry	Black	19.91 ± 1.66	18.39 ± 1.10	23.61 ± 1.71	0.13 ± 0.01	0.13 ± 0.00	72.37 ± 2.0	59.66 ± 4.90
	Nord Smelling	19.94 ± 1.61	18.76 ± 1.11	22.15 ± 1.60	0.21 ± 0.02	0.20 ± 0.01	70.21 ± 2.0	55.20 ± 2.10

Values are presented as mean ± standard deviation (SD) calculated from two independently prepared extracts obtained from a single pooled composite leaf sample for each cultivar. For each extract, measurements were performed in four technical replicate wells, and the mean value from the four wells was calculated separately. For α-amylase and α-glucosidase assays, concentration–response curves and the corresponding IC_50_ values were determined independently for each extract. The α-amylase and α-glucosidase assays were performed at preparation concentrations of 0.25, 0.50, and 1.00 mg/mL, with acarbose (0.05 and 0.20 mg/mL) used as the reference inhibitor. COX-1 and COX-2 inhibition values represent percentage inhibition determined at a final preparation concentration of 1.00 mg/mL; ibuprofen at a final concentration of 1.00 mg/mL was used as the reference inhibitor. The reported values were subsequently calculated from the two extraction-level results. Replicate wells were considered technical assay replicates, whereas the two extracts represent extraction replicates and not independent biological replicates. Accordingly, no inferential statistical comparisons among cultivar-specific composite samples were performed.

## Data Availability

The original data presented in the study are openly available in Wrocław University of Environmental and Life Sciences Repository at: https://doi.org/10.57755/3h51-vn76. URL: https://bazawiedzy.upwr.edu.pl/info/researc-data/UPWR59b36b3a3a164159a2b380a9a75edc36/ (accessed on 6 August 2026).

## Supplementary Materials

The following supporting information can be downloaded at https://www.mdpi.com/article/10.3390/molecules31193419/s1, Figure S1: Effects of raspberry leaf preparations from seven cultivars on HT29 human colorectal adenocarcinoma cell viability after 72 h of treatment. Cell viability was determined using the PrestoBlue® assay and expressed as the percentage of viable cells relative to untreated controls (100%). The chart includes the untreated control (K), DMSO solvent control, and raspberry leaf extract concentrations ranging from 50 to 600 µg/mL. For each cultivar, concentration series were prepared separately from two independently prepared preparations obtained from the corresponding pooled composite leaf sample. Each concentration was analyzed in four technical replicate wells for each extract. Values are presented as mean ± SD calculated from the two extraction-level mean values. Owing to the non-monotonic concentration–response pattern and substantial variability observed for HT29 cells, these data were not used for comparative evaluation of preparations effects or for IC_50_ estimation; Figure S2: Representative UPLC–PDA chromatogram of the ‘Polana’ red raspberry (*Rubus idaeus* L.) leaf extract recorded at 280 nm. Peak numbers correspond to compounds listed in Table S1 and are assigned according to increasing retention time. Only compounds giving clearly distinguishable chromatographic signals at 280 nm are labelled; Figure S3: Representative negative-ion UPLC–Q/TOF-MS/MS spectrum of quinic acid detected in raspberry leaf extract; Figure S4: Representative negative-ion UPLC–Q/TOF-MS/MS spectrum of gallic acid detected in raspberry leaf extract; Figure S5: Representative negative-ion UPLC–Q/TOF-MS/MS spectrum of caffeic acid detected in raspberry leaf extract; Figure S6: Representative negative-ion UPLC–Q/TOF-MS/MS spectrum of neochlorogenic acid detected in raspberry leaf extract; Figure S7: Representative negative-ion UPLC–Q/TOF-MS/MS spectrum of B-type procyanidin dimer isomer 1 detected in raspberry leaf extract; Figure S8: Representative negative-ion UPLC–Q/TOF-MS/MS spectrum of quercetin *O*-pentoside isomer 2 detected in raspberry leaf extract; Figure S9: Representative negative-ion UPLC–Q/TOF-MS/MS spectrum supporting the tentative annotation of a kaempferol glycoside derivative in raspberry leaf extract; Table S1: UPLC-MS/MS characterization of phenolic compounds detected in raspberry leaves, including retention times, accurate mass data, MS/MS fragmentation, and identification levels. Table S2: Calibration parameters of reference standards used for the quantitative and semi-quantitative determination of phenolic compounds in raspberry leaf extracts. Table S3: Spearman rank correlations between phenolic composition and biological activity endpoints across the seven analyzed cultivar-specific raspberry leaf composite samples (*n* = 7). Exact two-sided permutation-based *p*-values were calculated by exhaustive permutation and adjusted for multiple testing across all 77 correlations using the Benjamini–Hochberg false discovery rate procedure. Cell viability corresponds to the response measured at 600 μg/mL. Lower α-amylase and α-glucosidase IC_50_ values and lower cell viability indicate stronger effects, whereas higher COX-1 and COX-2 inhibition values indicate greater COX-inhibitory activity. FDR-adjusted q-values < 0.05 were considered statistically significant. Table S4: Estimated IC_50_ values and selectivity indices of raspberry leaf preparations toward colorectal cancer and non-malignant colon cells.
