# Supplementary material for: Polyphenolic Composition and Multifunctional Biological Properties of Raspberry Leaves: Variation Among Cultivar-Specific Samples
Source: Molecules. 2026 Sep 25;31(19):3419. doi: 10.3390/molecules31193419 (PMC13666087; doi:10.3390/molecules31193419)
Supplement: Supplementary file 1 [file molecules-31-03419-s001.zip › molecules-4517153-supplementary.pdf]

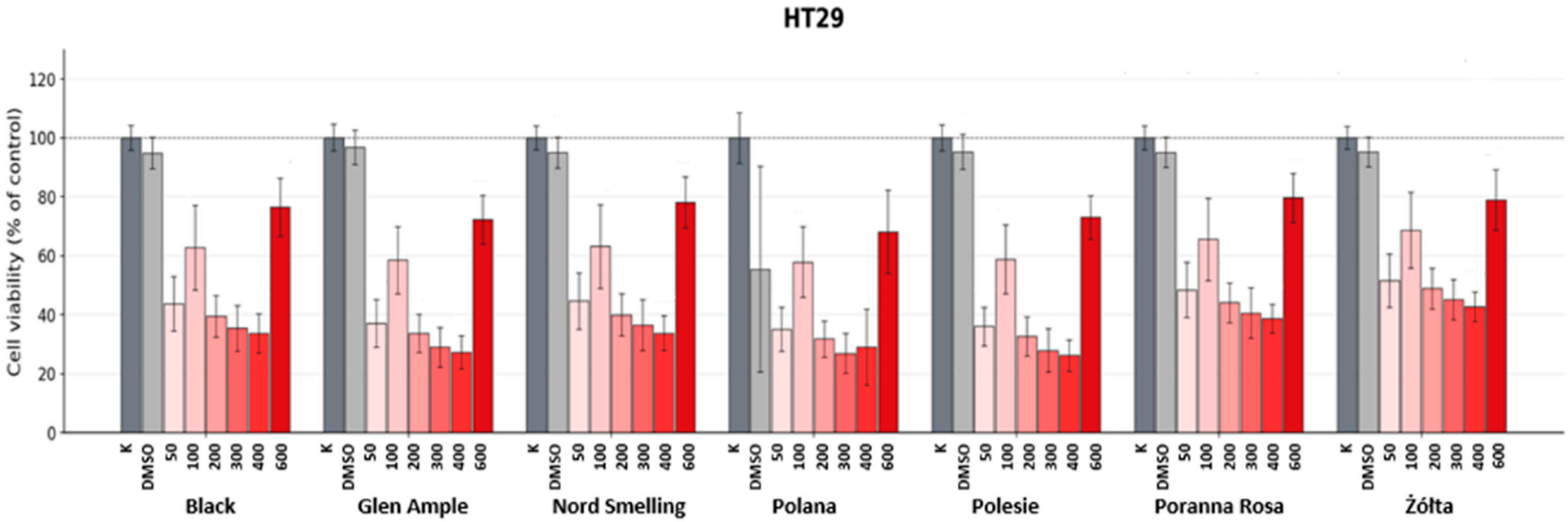

Figure. S1. Effects of raspberry leaf preparations from seven cultivars on HT29 human colorectal adenocarcinoma cell viability after 72 h of treatment. Cell viability was determined using the PrestoBlue® assay and expressed as the percentage of viable cells relative to untreated controls (100%). The chart includes the untreated control (K), DMSO solvent control, and raspberry leaf extract concentrations ranging from 50 to 600 µg/mL. For each cultivar, concentration series were prepared separately from two independently prepared preparations obtained from the corresponding pooled composite leaf sample. Each concentration was analyzed in four technical replicate wells for each extract. Values are presented as mean ± SD calculated from the two extraction-level mean values. Owing to the non-monotonic concentration–response pattern and substantial variability observed for HT29 cells, these data were not used for comparative evaluation of preparations effects or for IC_{50} estimation.

Figure S2. Representative UPLC–PDA chromatogram of the 'Polana' red raspberry (*Rubus idaeus* L.) leaf extract recorded at 280 nm. Peak numbers correspond to compounds listed in Table S1 and are assigned according to increasing retention time. Only compounds giving clearly distinguishable chromatographic signals at 280 nm are labelled

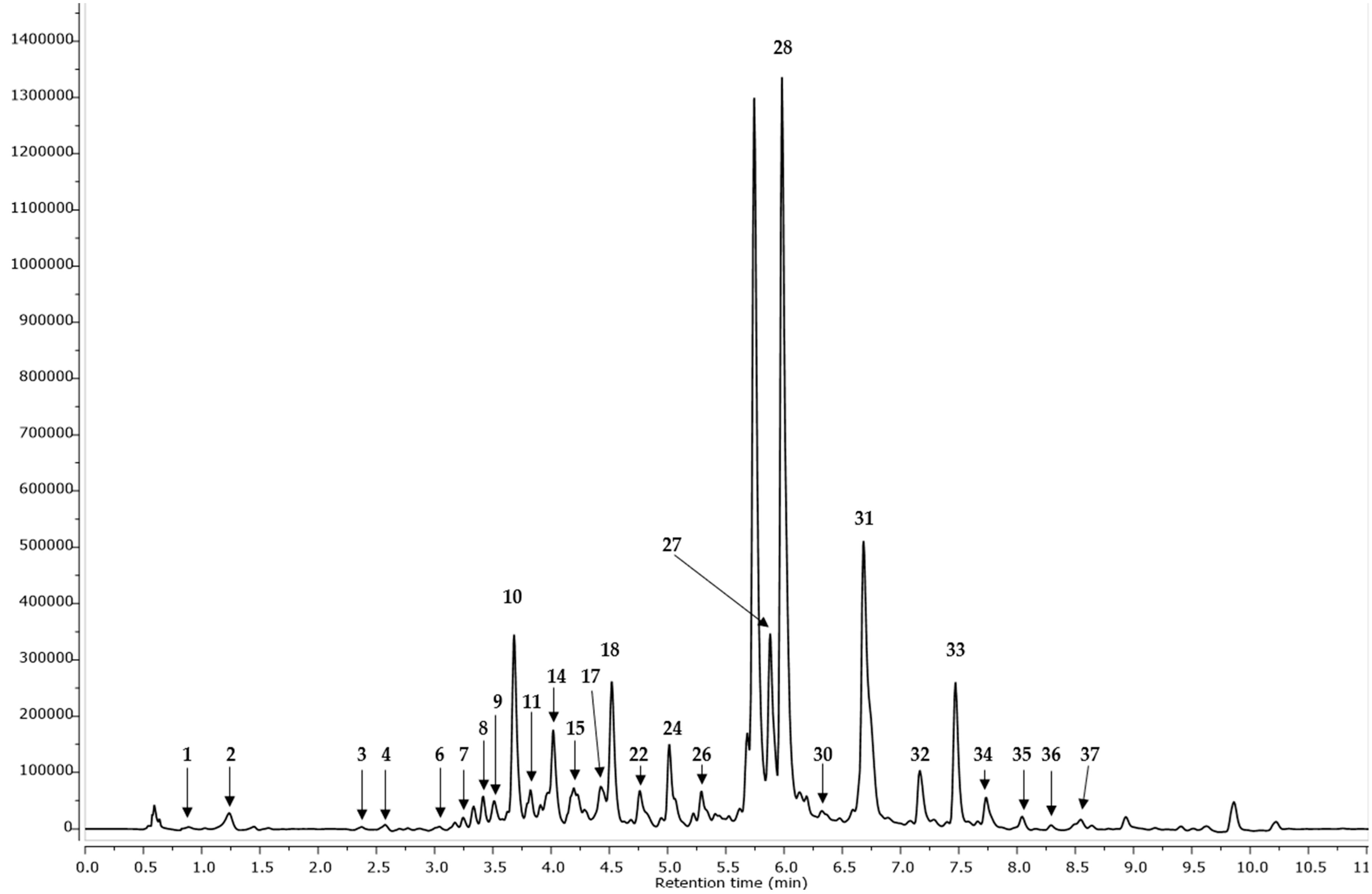

Figure S3. Representative negative-ion UPLC–Q/TOF-MS/MS spectrum of quinic acid detected in raspberry leaf extract

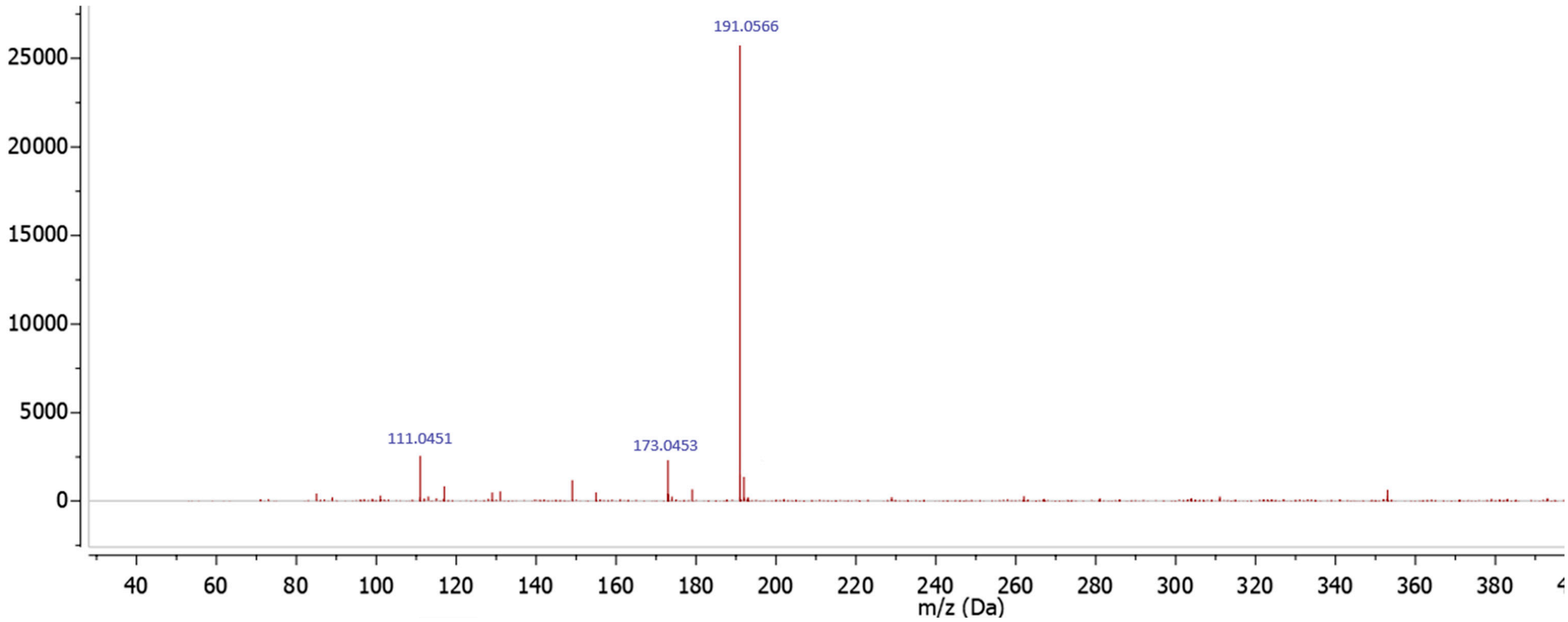

Figure S4. Representative negative-ion UPLC–Q/TOF-MS/MS spectrum of gallic acid detected in raspberry leaf extract

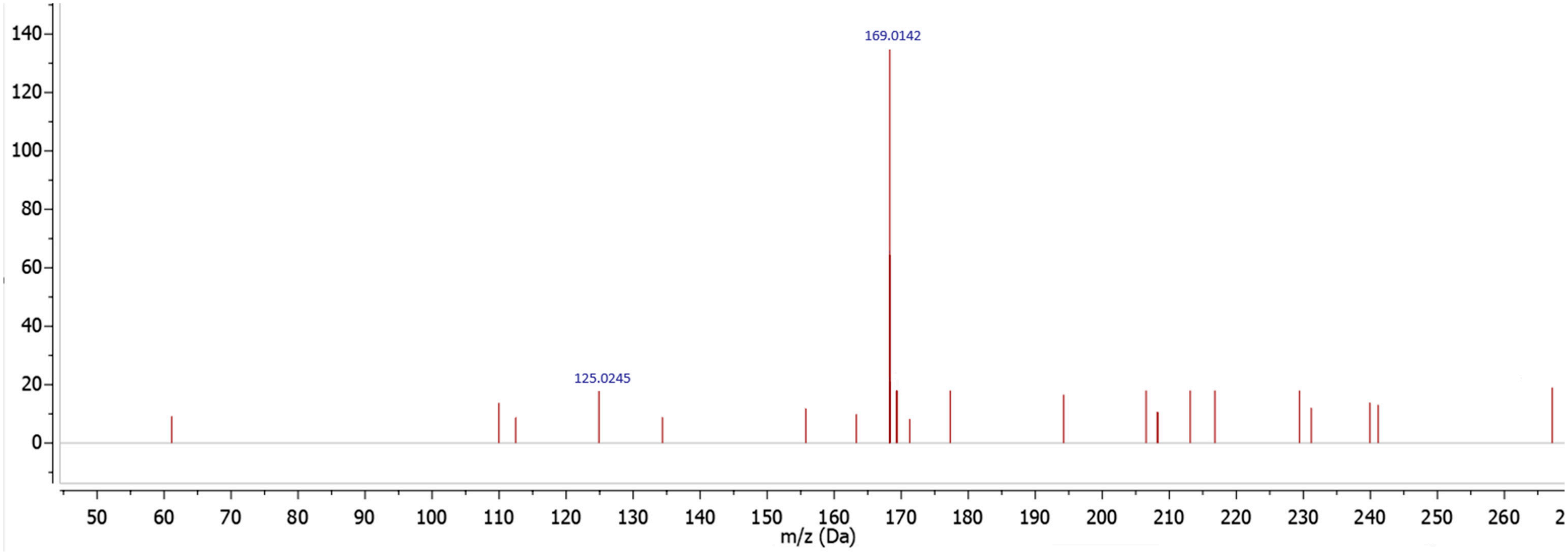

Figure S5. Representative negative-ion UPLC–Q/TOF-MS/MS spectrum of caffeic acid detected in raspberry leaf extract

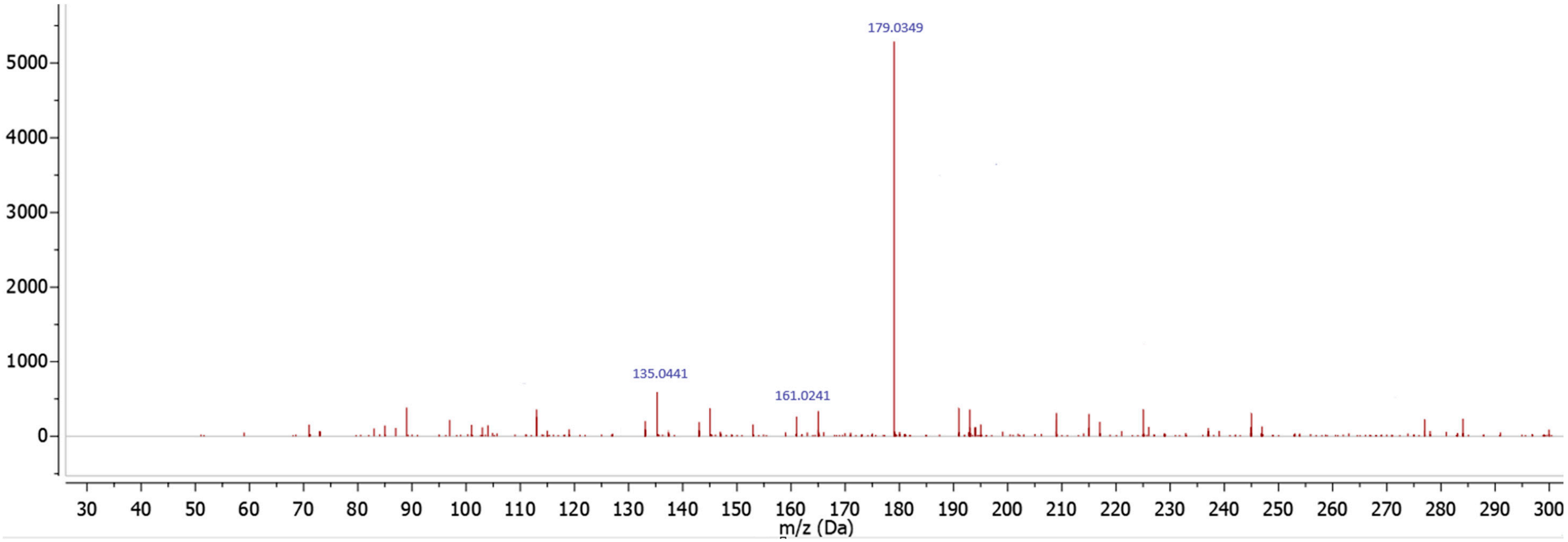

Figure S6. Representative negative-ion UPLC–Q/TOF-MS/MS spectrum of neochlorogenic acid detected in raspberry leaf extract

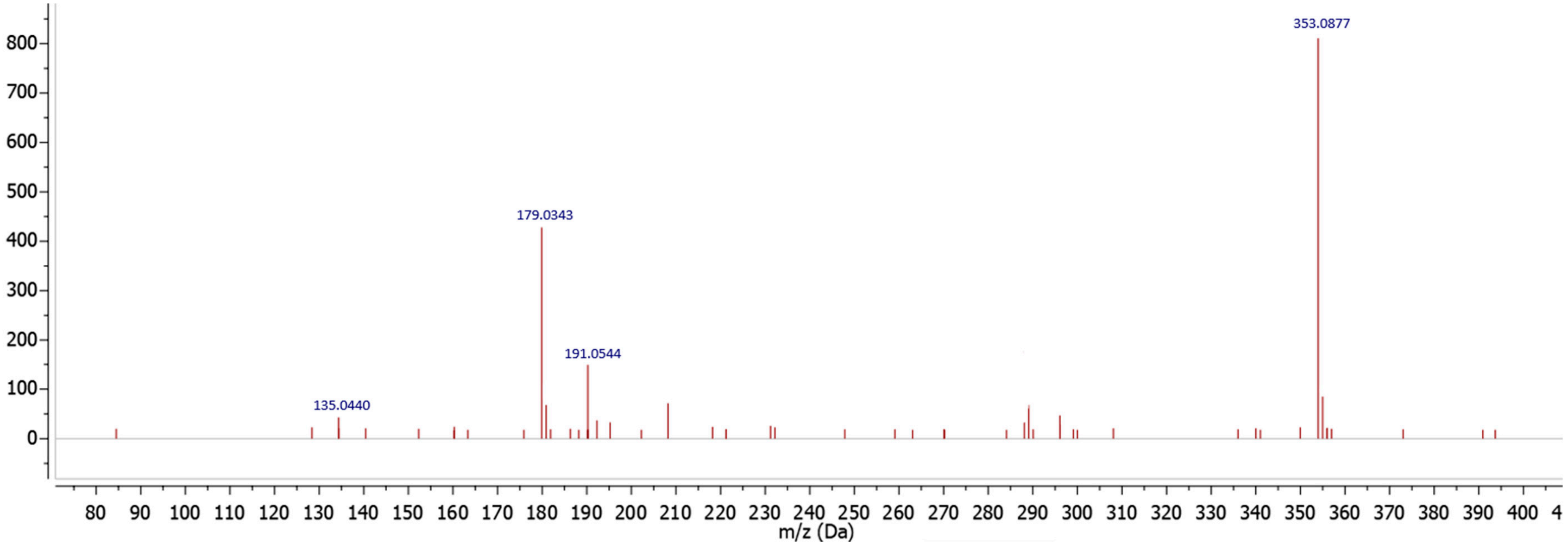

Figure S7. Representative negative-ion UPLC–Q/TOF-MS/MS spectrum of B-type procyanidin dimer isomer 1 detected in raspberry leaf extract

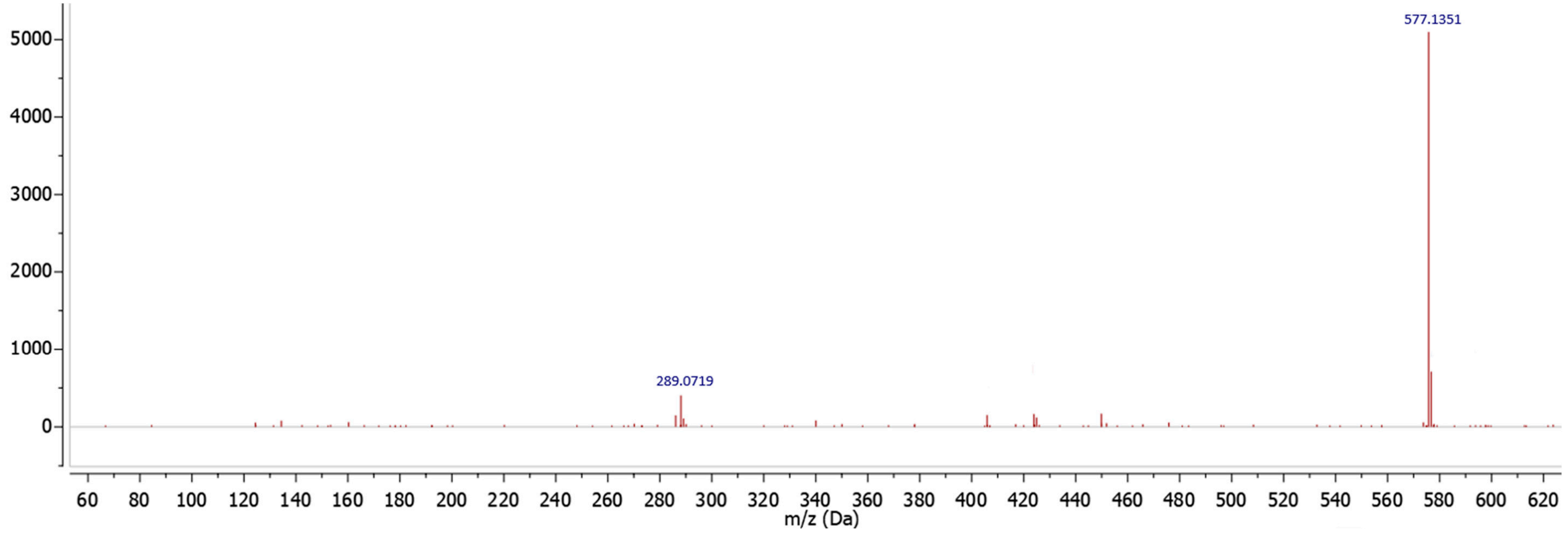

Figure S8. Representative negative-ion UPLC–Q/TOF-MS/MS spectrum of quercetin *O*-pentoside isomer 2 detected in raspberry leaf extract

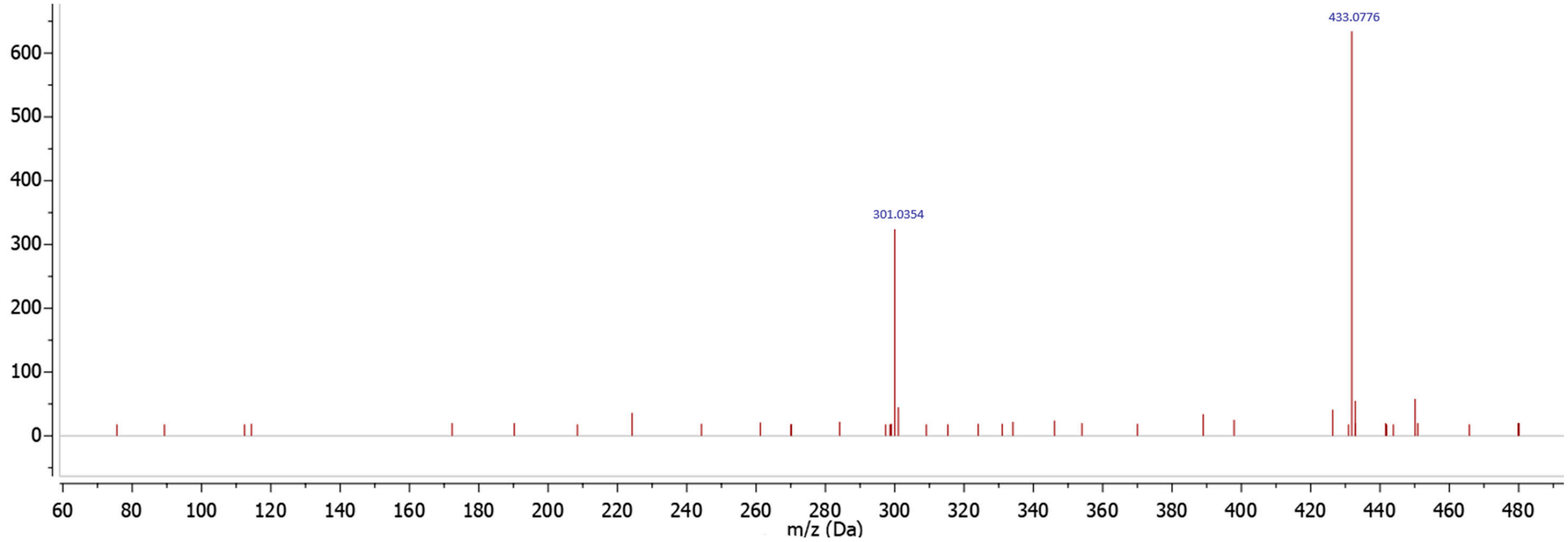

Figure S9. Representative negative-ion UPLC–Q/TOF-MS/MS spectrum supporting the tentative annotation of a kaempferol glycoside derivative in raspberry leaf extract

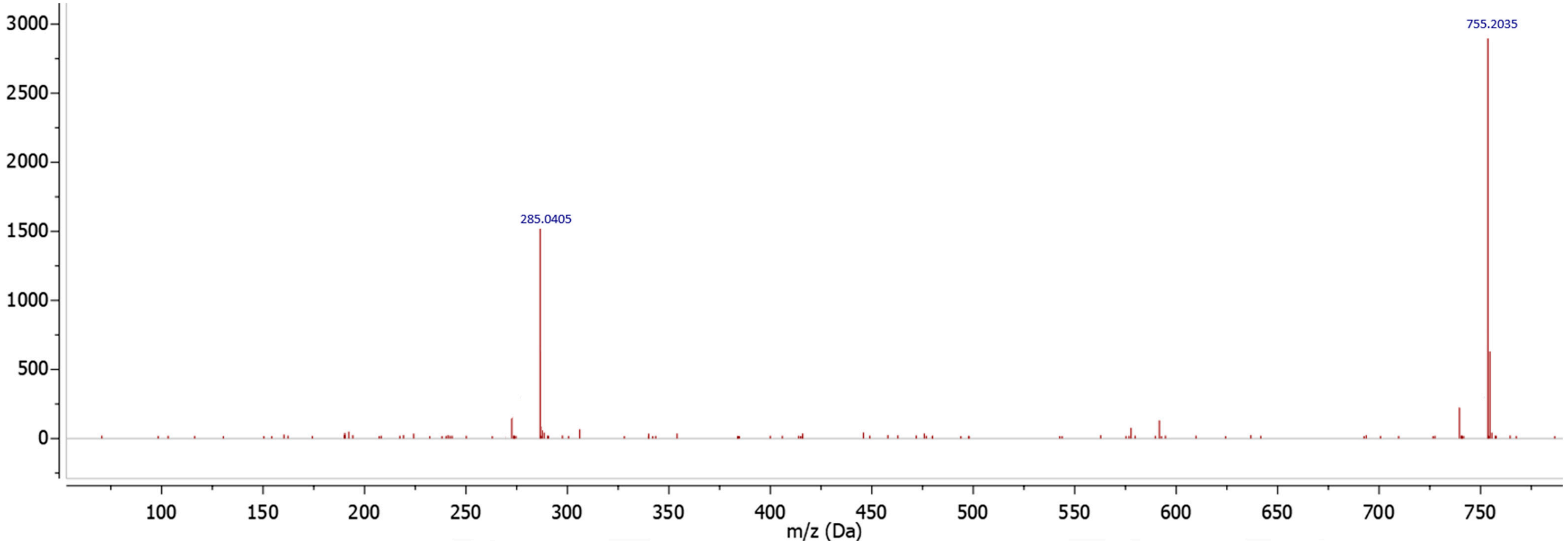

Table S1. UPLC-MS/MS characterization of phenolic compounds detected in raspberry leaves, including retention times, accurate mass data, MS/MS fragmentation, and identification levels

Peak No.	Class	Compound	Molecular formula	RT (min)	Ion	Theoretical m/z	Observed m/z	Error (ppm)	Major MS/MS fragments (m/z)	Identification level
1	Phenolic acids and derivatives	Quinic acid	$C_7H_{12}O_6$	0.90	$[M{-}H]^-$	191.0561	191.0566	2.56	173.0453. 111.0451	Level 2
2	Phenolic acids and derivatives	Gallic acid *O*-hexoside	$C_{13}H_{16}O_{10}$	1.23	$[M{-}H]^-$	331.0671	331.0670	-0.21	169.0141	Level 2
3	Phenolic acids and derivatives	Gallic acid	$C_7H_6O_5$	2.37	$[M{-}H]^-$	169.0142	169.0142	-0.28	125.0245	Level 1 – confirmed identification
4	Phenolic acids and derivatives	Neochlorogenic acid	$C_{16}H_{18}O_9$	2.58	$[M{-}H]^-$	353.0878	353.0877	-0.30	191.0554. 179.0343. 135.0440	Level 1 – confirmed identification
5	Phenolic acids and derivatives	Ellagic acid *O*-pentoside	$C_{19}H_{14}O_{12}$	2.88	$[M{-}H]^-$	433.0412	433.0409	-0.81	300.9998	Level 2
6	Phenolic acids and derivatives	Caffeic acid	$C_9H_8O_4$	3.02	$[M{-}H]^-$	179.0350	179.0349	-0.46	135.0441. 161.0241	Level 1 – confirmed identification
7	Phenolic acids and derivatives	Caffeic acid *O*-hexoside isomer 1	$C_{15}H_{18}O_9$	3.25	$[M{-}H]^-$	341.0878	341.0877	-0.31	179.0341. 161.0244	Level 2
8	Phenolic acids and derivatives	Caffeic acid *O*-hexoside isomer 2	$C_{15}H_{18}O_9$	3.42	$[M{-}H]^-$	341.0878	341.0877	-0.31	179.0340. 161.0240	Level 2
9	Phenolic acids and derivatives	Chlorogenic acid	$C_{16}H_{18}O_9$	3.51	$[M{-}H]^-$	353.0878	353.0878	-0.02	191.0551. 179.0341. 135.0440	Level 2
11	Phenolic acids and derivatives	Caffeic acid *O*-hexoside isomer 3	$C_{15}H_{18}O_9$	3.65	$[M{-}H]^-$	341.0878	341.0877	-0.31	179.0341. 161.0240	Level 2
12	Phenolic acids and derivatives	Caffeic acid *O*-hexoside isomer 4	$C_{15}H_{18}O_9$	3.68	$[M{-}H]^-$	341.0878	341.0879	0.28	179.0352. 135.0442	Level 2
13	Phenolic acids and derivatives	Caffeoylthreonic acid isomer	$C_{13}H_{14}O_8$	3.71	$[M{-}H]^-$	297.0616	297.0614	-0.64	179.0350. 135.0291	Level 2
14	Phenolic acids and derivatives	Caffeoylquinic acid isomer	$C_{16}H_{18}O_9$	4.02	$[M{-}H]^-$	353.0878	353.0877	-0.30	191.0551	Level 2
30	Phenolic acids and derivatives	Ellagic acid acetyl pentoside isomer 1	$C_{21}H_{16}O_{13}$	6.32	$[M{-}H]^-$	475.0518	475.0525	1.44	300.9999	Level 2
31	Phenolic acids and derivatives	Ellagic acid acetyl pentoside isomer 2	$C_{21}H_{16}O_{13}$	6.69	$[M{-}H]^-$	475.0518	475.0527	1.86	300.9998	Level 2

35	Phenolic acids and derivatives	p-Coumaroyl-caffeoylquinic acid isomer	$C_{25}H_{24}O_{11}$	8.05	$[M-H]^-$	499.1246	499.1247	0.23	353.0877. 337.0930. 191.0555. 179.0345	Level 2
36	Phenolic acids and derivatives	Caffeoylglucose derivative	$C_{24}H_{24}O_{11}$	8.31	$[M-H]^-$	487.1246	487.1245	-0.17	341.0877. 179.0343. 161.0244	Level 3 – tentative compound class
37	Phenolic acids and derivatives	Dicaffeoylquinic acid isomer	$C_{25}H_{24}O_{12}$	8.54	$[M-H]^-$	515.1195	515.1192	-0.58	353.0877. 191.0555. 161.0240	Level 2
10	Flavan-3-ols and procyanidins	Epicatechin	$C_{15}H_{14}O_6$	3.59	$[M-H]^-$	289.0718	289.0719	0.48	245.0820. 205.0505. 179.0350. 137.0239	Level 2
16	Flavan-3-ols and procyanidins	B-type procyanidin dimer isomer 1	$C_{30}H_{26}O_{12}$	4.25	$[M-H]^-$	577.1351	577.1350	-0.26	289.0719	Level 2
17	Flavan-3-ols and procyanidins	B-type procyanidin dimer isomer 2	$C_{30}H_{26}O_{12}$	4.45	$[M-H]^-$	577.1351	577.1350	-0.26	289.0719	Level 2
18	Flavan-3-ols and procyanidins	B-type procyanidin dimer isomer 3	$C_{30}H_{26}O_{12}$	4.52	$[M-H]^-$	577.1351	577.1350	-0.26	289.0719	Level 2
19	Flavan-3-ols and procyanidins	B-type procyanidin dimer isomer 4	$C_{30}H_{26}O_{12}$	4.64	$[M-H]^-$	577.1351	577.1350	-0.26	425.0878. 289.0719	Level 2
21	Flavan-3-ols and procyanidins	B-type procyanidin trimer	$C_{45}H_{38}O_{18}$	4.71	$[M-H]^-$	865.1985	865.1986	0.07	577.1350. 289.0718	Level 2
22	Flavan-3-ols and procyanidins	A-type procyanidin trimer	$C_{45}H_{36}O_{18}$	4.77	$[M-H]^-$	863.1829	863.1830	0.13	575.1195. 287.0560	Level 2
24	Flavan-3-ols and procyanidins	Catechin	$C_{15}H_{14}O_6$	5.02	$[M-H]^-$	289.0718	289.0718	0.13	245.0820	Level 1 – confirmed identification
25	Flavan-3-ols and procyanidins	Catechin gallate	$C_{22}H_{18}O_{10}$	5.22	$[M-H]^-$	441.0827	441.0828	0.18	289.0719	Level 2
15	Flavonols	Kaempferol glycoside derivative	$C_{33}H_{40}O_{20}$	4.18	$[M-H]^-$	755.2040	755.2035	-0.68	285.0405	Level 3 – tentative compound class
20	Flavonols	Quercetin *O*-rutinoside	$C_{27}H_{30}O_{16}$	4.69	$[M-H]^-$	609.1461	609.1455	-1.00	301.0355	Level 2
26	Flavonols	Kaempferol pentosyl-hexuronide	$C_{26}H_{26}O_{16}$	5.35	$[M-H]^-$	593.1148	593.1149	0.15	461.0725. 417.0826. 285.0406	Level 2
27	Flavonols	Quercetin *O*-pentoside isomer 1	$C_{20}H_{18}O_{11}$	5.92	$[M-H]^-$	433.0776	433.0777	0.15	301.0354	Level 2
28	Flavonols	Quercetin *O*-pentoside isomer 2	$C_{20}H_{18}O_{11}$	5.99	$[M-H]^-$	433.0776	433.0776	-0.08	301.0354	Level 2

29	Flavonols	Quercetin HMG-hexoside	$C_{27}H_{28}O_{16}$	6.24	$[M-H]^-$	607.1305	607.1301	-0.59	301.0353	Level 2
32	Flavonols	Quercetin *O*-pentoside isomer 3	$C_{20}H_{18}O_{11}$	7.18	$[M-H]^-$	433.0776	433.0777	0.15	301.0355	Level 2
33	Flavonols	Kaempferol *O*-hexuronide	$C_{21}H_{18}O_{12}$	7.48	$[M-H]^-$	461.0725	461.0726	0.11	285.0405	Level 2
34	Flavonols	Kaempferol *O*-pentoside	$C_{20}H_{18}O_{10}$	7.74	$[M-H]^-$	417.0827	417.0827	-0.05	285.0406	Level 2
23	Flavones	Apigenin *O*-pentoside	$C_{20}H_{18}O_{9}$	4.95	$[M-H]^-$	401.0878	401.0877	-0.26	269.0456	Level 2

Compounds were characterized based on retention time (RT), accurate mass measurements, and MS/MS fragmentation patterns. Identification levels were assigned according to the available analytical evidence: Level 1, confirmed identification based on comparison with an authentic standard; Level 2, putatively annotated compound based on accurate mass and MS/MS fragmentation data supported by literature; and Level 3, tentative compound class assignment based on accurate mass and characteristic MS/MS fragmentation. Mass error was calculated as the difference between the observed and theoretical m/z values and is expressed in parts per million (ppm). RT, retention time; MS/MS, tandem mass spectrometry.

Table S2. Calibration parameters of reference standards used for the quantitative and semi-quantitative determination of phenolic compounds in raspberry leaf extracts.

Reference standard	Calibration range (ppm)	Calibration equation	R^2	LOD (ppm)	LOQ (ppm)
3-Caffeoylquinic acid	LOQ-335	y = 19835x + 31727	0.9998	0.09	0.3
Gallic acid	LOQ-385	y = 1222.5x - 1972.7	0.9999	0.13	0.4
Ellagic acid	LOQ-385	y = 52815x + 38406	0.9997	0.09	0.2
Caffeic acid	LOQ-385	y = 17431x + 40114	0.9997	0.03	0.1
(+)-Catechin	LOQ-385	y = 1565.9x + 2243	0.9999	0.17	0.6
Procyanidin A2	LOQ-225	y = 9484.1x - 6770.5	0.9999	0.06	0.2
Procyanidin B2	LOQ-260	y = 19665x + 9754.3	0.9998	0.22	0.7
Quercetin 3-O-glucoside	LOQ-180	y = 11923x + 8188	0.9999	0.03	0.1
Kaempferol 3-O-glucoside	LOQ-110	y = 12057x - 1922.4	0.9999	0.07	0.2
Apigenin 7-O-glucoside	LOQ-115	y = 735.47x -394.17	0.9999	0.03	0.1

Abbreviations: LOD, limit of detection; LOQ, limit of quantification. Calibration curves were constructed using external reference standards. The lower limit of each calibration range corresponded to the respective LOQ. Calibration ranges, LOD, and LOQ values are expressed in ppm.

Table S3. Spearman rank correlations between phenolic composition and biological activity endpoints across the seven analyzed cultivar-specific raspberry leaf composite samples (n = 7). Exact two-sided permutation-based p-values were calculated by exhaustive permutation and adjusted for multiple testing across all 77 correlations using the Benjamini–Hochberg false discovery rate procedure. Cell viability corresponds to the response measured at 600 µg/mL. Lower α-amylase and α-glucosidase IC_{50} values and lower cell viability indicate stronger effects, whereas higher COX-1 and COX-2 inhibition values indicate greater COX-inhibitory activity. FDR-adjusted q-values < 0.05 were considered statistically significant.

Phenolic variable	Biological endpoint	Spearman's ϱ	Exact two-sided p-value	FDR-adjusted q-value
Phenolic acids and derivatives	ABTS	0.8571	0.023809524	0.091666667
Phenolic acids and derivatives	DPPH	0.6071	0.166666667	0.329059829
Phenolic acids and derivatives	FRAP	0.9643	0.002777778	0.019444444
Phenolic acids and derivatives	α-amylase IC_{50}	-0.4505	0.315873016	0.506712963
Phenolic acids and derivatives	α-glucosidase IC_{50}	-0.4144	0.357142857	0.528846154
Phenolic acids and derivatives	COX-1 inhibition	-0.6786	0.109523810	0.234259259
Phenolic acids and derivatives	COX-2 inhibition	-0.3929	0.395634921	0.553888889
Phenolic acids and derivatives	LOVO viability (600 µg/mL)	-0.9286	0.006746032	0.037103175
Phenolic acids and derivatives	LOVO-DX viability (600 µg/mL)	-0.9286	0.006746032	0.037103175
Phenolic acids and derivatives	SW48 viability (600 µg/mL)	-0.9286	0.006746032	0.037103175
Phenolic acids and derivatives	CCD 841 CoTr viability (600 µg/mL)	-0.7143	0.088095238	0.218817204
Ellagic acid derivatives	ABTS	0.4286	0.353571429	0.528846154
Ellagic acid derivatives	DPPH	0.4643	0.302380952	0.495390071
Ellagic acid derivatives	FRAP	0.4286	0.353571429	0.528846154
Ellagic acid derivatives	α-amylase IC_{50}	-0.2883	0.537301587	0.701775956
Ellagic acid derivatives	α-glucosidase IC_{50}	-0.1982	0.669841270	0.758496732
Ellagic acid derivatives	COX-1 inhibition	-0.2500	0.594841270	0.726851852
Ellagic acid derivatives	COX-2 inhibition	-0.3929	0.395634921	0.553888889
Ellagic acid derivatives	LOVO viability (600 µg/mL)	-0.4643	0.302380952	0.495390071
Ellagic acid derivatives	LOVO-DX viability (600 µg/mL)	-0.4643	0.302380952	0.495390071
Ellagic acid derivatives	SW48 viability (600 µg/mL)	-0.4643	0.302380952	0.495390071
Ellagic acid derivatives	CCD 841 CoTr viability (600 µg/mL)	-0.2857	0.555952381	0.701775956
Flavan-3-ols	ABTS	0.9643	0.002777778	0.019444444
Flavan-3-ols	DPPH	0.8214	0.034126984	0.109490741
Flavan-3-ols	FRAP	0.9643	0.002777778	0.019444444
Flavan-3-ols	α-amylase IC_{50}	-0.2342	0.623015873	0.726851852
Flavan-3-ols	α-glucosidase IC_{50}	-0.2342	0.623015873	0.726851852
Flavan-3-ols	COX-1 inhibition	-0.7857	0.048015873	0.142200855
Flavan-3-ols	COX-2 inhibition	-0.5714	0.200000000	0.366666667

Flavan-3-ols	LOVO viability (600 μg/mL)	-1.0000	0.000396825	0.005092593
Flavan-3-ols	LOVO-DX viability (600 μg/mL)	-1.0000	0.000396825	0.005092593
Flavan-3-ols	SW48 viability (600 μg/mL)	-1.0000	0.000396825	0.005092593
Flavan-3-ols	CCD 841 CoTr viability (600 μg/mL)	-0.8214	0.034126984	0.109490741
Procyanidins	ABTS	0.8571	0.023809524	0.091666667
Procyanidins	DPPH	0.6786	0.109523810	0.234259259
Procyanidins	FRAP	0.8571	0.023809524	0.091666667
Procyanidins	α-amylase IC_{50}	-0.1081	0.819047619	0.852252252
Procyanidins	α-glucosidase IC_{50}	-0.0901	0.859523810	0.870833333
Procyanidins	COX-1 inhibition	-0.6071	0.166666667	0.329059829
Procyanidins	COX-2 inhibition	-0.3214	0.497619048	0.684226190
Procyanidins	LOVO viability (600 μg/mL)	-0.8929	0.012301587	0.055718954
Procyanidins	LOVO-DX viability (600 μg/mL)	-0.8929	0.012301587	0.055718954
Procyanidins	SW48 viability (600 μg/mL)	-0.8929	0.012301587	0.055718954
Procyanidins	CCD 841 CoTr viability (600 μg/mL)	-0.7143	0.088095238	0.218817204
Flavonols	ABTS	0.7143	0.088095238	0.218817204
Flavonols	DPPH	0.7500	0.066269841	0.188991770
Flavonols	FRAP	0.5714	0.200000000	0.366666667
Flavonols	α-amylase IC_{50}	0.0360	0.950793651	0.950793651
Flavonols	α-glucosidase IC_{50}	0.0901	0.859523810	0.870833333
Flavonols	COX-1 inhibition	-0.3929	0.395634921	0.553888889
Flavonols	COX-2 inhibition	-0.5357	0.235714286	0.422093023
Flavonols	LOVO viability (600 μg/mL)	-0.6786	0.109523810	0.234259259
Flavonols	LOVO-DX viability (600 μg/mL)	-0.6786	0.109523810	0.234259259
Flavonols	SW48 viability (600 μg/mL)	-0.6786	0.109523810	0.234259259
Flavonols	CCD 841 CoTr viability (600 μg/mL)	-0.4286	0.353571429	0.528846154
Flavones	ABTS	-0.1429	0.782539683	0.825418569
Flavones	DPPH	-0.1429	0.782539683	0.825418569
Flavones	FRAP	-0.2857	0.555952381	0.701775956
Flavones	α-amylase IC_{50}	0.7388	0.070634921	0.194246032
Flavones	α-glucosidase IC_{50}	0.6487	0.122222222	0.254354354
Flavones	COX-1 inhibition	0.2143	0.661507937	0.758496732
Flavones	COX-2 inhibition	0.2857	0.555952381	0.701775956
Flavones	LOVO viability (600 μg/mL)	0.1786	0.713095238	0.773356808
Flavones	LOVO-DX viability (600 μg/mL)	0.1786	0.713095238	0.773356808

Flavones	SW48 viability (600 μg/mL)	0.1786	0.713095238	0.773356808
Flavones	CCD 841 CoTr viability (600 μg/mL)	0.2857	0.555952381	0.701775956
Total polyphenols	ABTS	0.9643	0.002777778	0.019444444
Total polyphenols	DPPH	0.8214	0.034126984	0.109490741
Total polyphenols	FRAP	0.9643	0.002777778	0.019444444
Total polyphenols	α-amylase IC_{50}	-0.2342	0.623015873	0.726851852
Total polyphenols	α-glucosidase IC_{50}	-0.2342	0.623015873	0.726851852
Total polyphenols	COX-1 inhibition	-0.7857	0.048015873	0.142200855
Total polyphenols	COX-2 inhibition	-0.5714	0.200000000	0.366666667
Total polyphenols	LOVO viability (600 μg/mL)	-1.0000	0.000396825	0.005092593
Total polyphenols	LOVO-DX viability (600 μg/mL)	-1.0000	0.000396825	0.005092593
Total polyphenols	SW48 viability (600 μg/mL)	-1.0000	0.000396825	0.005092593
Total polyphenols	CCD 841 CoTr viability (600 μg/mL)	-0.8214	0.034126984	0.109490741

Table S4. Estimated IC_{50} values and selectivity indices of raspberry leaf preparations toward colorectal cancer and non-malignant colon cells

Sample	LOVO IC_{50}	SI vs LOVO	LOVO-DX IC_{50}	SI vs LOVO-DX	SW48 IC_{50}	SI vs SW48	CCD IC_{50}
Polana	426.7	>1.41	536.5	>1.12	443.4	>1.35	>600
Polesie	432.9	>1.39	542.1	>1.11	455.6	>1.32	>600
Glen Ample	441.6	>1.36	553.0	>1.08	467.9	>1.28	>600
Żółta	552.9	>1.09	>600	n.d.	>600	n.d.	>600
Poranna Rosa	511.5	>1.17	594.8	>1.01	588.9	>1.02	>600
Nord Smelling	479.7	>1.25	581.3	>1.03	549.0	>1.09	>600
Black	477.8	>1.26	574.7	>1.04	533.1	>1.13	>600

IC_{50} values are expressed as μg/mL of purified lyophilized raspberry leaf preparation. IC_{50} values were estimated by linear interpolation between adjacent tested concentrations encompassing 50% cell viability. Values >600 μg/mL indicate that 50% viability was not reached within the tested concentration range. SI was calculated as IC_{50}(CCD 841 CoTr)/IC_{50}(cancer cell line). Because IC_{50}(CCD 841 CoTr) was >600 μg/mL for all preparations, SI values are reported as lower-bound estimates. n.d., not determined because IC_{50} was >600 μg/mL for both cell lines.
